# Inter-laboratory assessment of different digital PCR platforms for quantification of human cytomegalovirus DNA

**DOI:** 10.1007/s00216-017-0206-0

**Published:** 2017-01-26

**Authors:** Jernej Pavšič, Alison Devonshire, Andrej Blejec, Carole A. Foy, Fran Van Heuverswyn, Gerwyn M. Jones, Heinz Schimmel, Jana Žel, Jim F. Huggett, Nicholas Redshaw, Maria Karczmarczyk, Erkan Mozioğlu, Sema Akyürek, Müslüm Akgöz, Mojca Milavec

**Affiliations:** 1 Department of Biotechnology and Systems Biology, National Institute of Biology, Večna pot 111, 1000 Ljubljana, Slovenia; 2 Jožef Stefan International Postgraduate School, Jamova 39, 1000 Ljubljana, Slovenia; 3 Molecular and Cell Biology, LGC Ltd., Queens Road, Teddington, Middlesex TW11 0LY UK; 4 European Commission, Joint Research Centre (JRC), Directorate F. Retieseweg 111, 2440 Geel, Belgium; 5 School of Biosciences and Medicine, Faculty of Health and Medical Science, University of Surrey, Guildford, Surrey GU2 7XH UK; 6 Bioanalysis Laboratory, National Metrology Institute of Turkey, (TUBITAK UME), PO Box 54, 41470 Gebze, Kocaeli Turkey

**Keywords:** Digital PCR, DNA quantification, Inter-laboratory assessment, Human cytomegalovirus, Virus reference materials

## Abstract

Quantitative PCR (qPCR) is an important tool in pathogen detection. However, the use of different qPCR components, calibration materials and DNA extraction methods reduces comparability between laboratories, which can result in false diagnosis and discrepancies in patient care. The wider establishment of a metrological framework for nucleic acid tests could improve the degree of standardisation of pathogen detection and the quantification methods applied in the clinical context. To achieve this, accurate methods need to be developed and implemented as reference measurement procedures, and to facilitate characterisation of suitable certified reference materials. Digital PCR (dPCR) has already been used for pathogen quantification by analysing nucleic acids. Although dPCR has the potential to provide robust and accurate quantification of nucleic acids, further assessment of its actual performance characteristics is needed before it can be implemented in a metrological framework, and to allow adequate estimation of measurement uncertainties. Here, four laboratories demonstrated reproducibility (expanded measurement uncertainties below 15%) of dPCR for quantification of DNA from human cytomegalovirus, with no calibration to a common reference material. Using whole-virus material and extracted DNA, an intermediate precision (coefficients of variation below 25%) between three consecutive experiments was noted. Furthermore, discrepancies in estimated mean DNA copy number concentrations between laboratories were less than twofold, with DNA extraction as the main source of variability. These data demonstrate that dPCR offers a repeatable and reproducible method for quantification of viral DNA, and due to its satisfactory performance should be considered as candidate for reference methods for implementation in a metrological framework.

**Electronic supplementary material:**

The online version of this article (doi:10.1007/s00216-017-0206-0) contains supplementary material, which is available to authorized users.

## Introduction

Nucleic acid amplification-based tests offer an important method for rapid and reliable diagnosis of infectious diseases. Quantitative polymerase chain reaction (qPCR) has become a valuable tool for routine microbiology testing, as it allows rapid, specific and sensitive detection and quantification of viral and bacterial nucleic acids in a broad range of samples [1–3]. However, clinical laboratories often use different qPCR platforms, different commercial or in-house calibration materials (e.g. secondary reference materials), and different PCR components, which can in turn result in significant variability of the reported quantitative (i.e. concentration of pathogen) and qualitative (i.e. presence or absence of pathogen) data between laboratories [2, 4, 5]. Lack of agreement in reported quantitative data hampers the definition of generally accepted clinical thresholds for initiation or termination of anti-pathogen therapies, while disparities in qualitative data can result in misdiagnosis of a disease [4].

The establishment of a reference measurement system for nucleic acid amplification-based tests through the development of reference measurement procedures and suitable reference materials would facilitate standardisation of quantitative and qualitative measurements within the international clinical community. Metrological traceability of end-user measurements to reference measurement procedures, that have been used to value assign reference materials of higher metrological order, could enhance equivalence of measurements over time and space [6]. Suitable reference materials with assigned values that are traceable to the International System of Units (SI units) or to other internationally accepted standards (e.g. international units; IU) would facilitate accurate and reproducible characterisation of reference materials at a lower level in the calibration hierarchy and/or of calibrators produced by different manufacturers [6, 7]. This should, in turn, increase agreement between end-user quantitative measurements and improve assessment of the analytical performance characteristics of quantitative and qualitative nucleic acid amplification-based methods [4]. Additionally, reference measurement procedures that have well-defined measurement uncertainty and are independent of external calibrators are needed to provide reproducible, precise and accurate enumeration and characterisation of reference materials of higher metrological order, including assessment of their homogeneity and stability over time [4, 6].

For a small number of more important viruses, reference materials that are composed of either cultured whole virus (World Health Organisation, WHO International Reference Materials) or plasmids (National Institute of Standards and Technology; NIST) have already been developed, traceable to arbitrary assigned international units (IU, by WHO), or SI units in terms of plasmid copy numbers (NIST) [8–10]. However, for the great majority of viruses and bacteria, suitable reference materials for calibration purposes have not been developed yet, while with human cytomegalovirus (HCMV), there remains discordance for the viral loads between the different commercial reference materials [7]. As the value assignments of the existing WHO reference materials were performed in collaborative studies using various qPCR-based methods calibrated against arbitrary assigned external calibrators [8], there is a challenge as neither the qPCR method nor the external calibrator are anchored in an unbiased way to a uniform reference. Consequently, value assignment of different batches of calibrators and in vitro diagnostics is challenging; a factor that could be rectified if a suitable reference method was available and provided the intermediate reference materials and calibrators are suited to allow unbiased value transfer.

Digital (d)PCR has the potential to provide accurate and robust end-point measurements of nucleic acid copy number concentration and is therefore a promising candidate for a reference measurement procedure. It has been demonstrated that dPCR is more resistant (although not completely insensitive) to PCR inhibitors than qPCR [11, 12]; hence, it is expected to deliver more robust and accurate nucleic acid quantification than qPCR [13]. However, the reported over-estimation and under-estimation of nucleic acid copy number concentration indicate the need for optimisation of the analytical process of dPCR, so that homogenous distributions of nucleic acids can be achieved during partitioning, along with their successful amplification during the PCR cycling [14, 15]. dPCR has already been used for different applications, such as quantification of viruses and bacteria [16–18], quantification of virus reference materials [7, 19, 20] and value assignment of certified reference materials that consist of plasmid DNA [10, 14, 21]. However, as with every novel technology, comprehensive inter-laboratory assessments with different dPCR platforms need to be conducted to confirm the suitability of dPCR-based methods for value assignment of reference materials for viruses and bacteria.

**Fig. 1:**
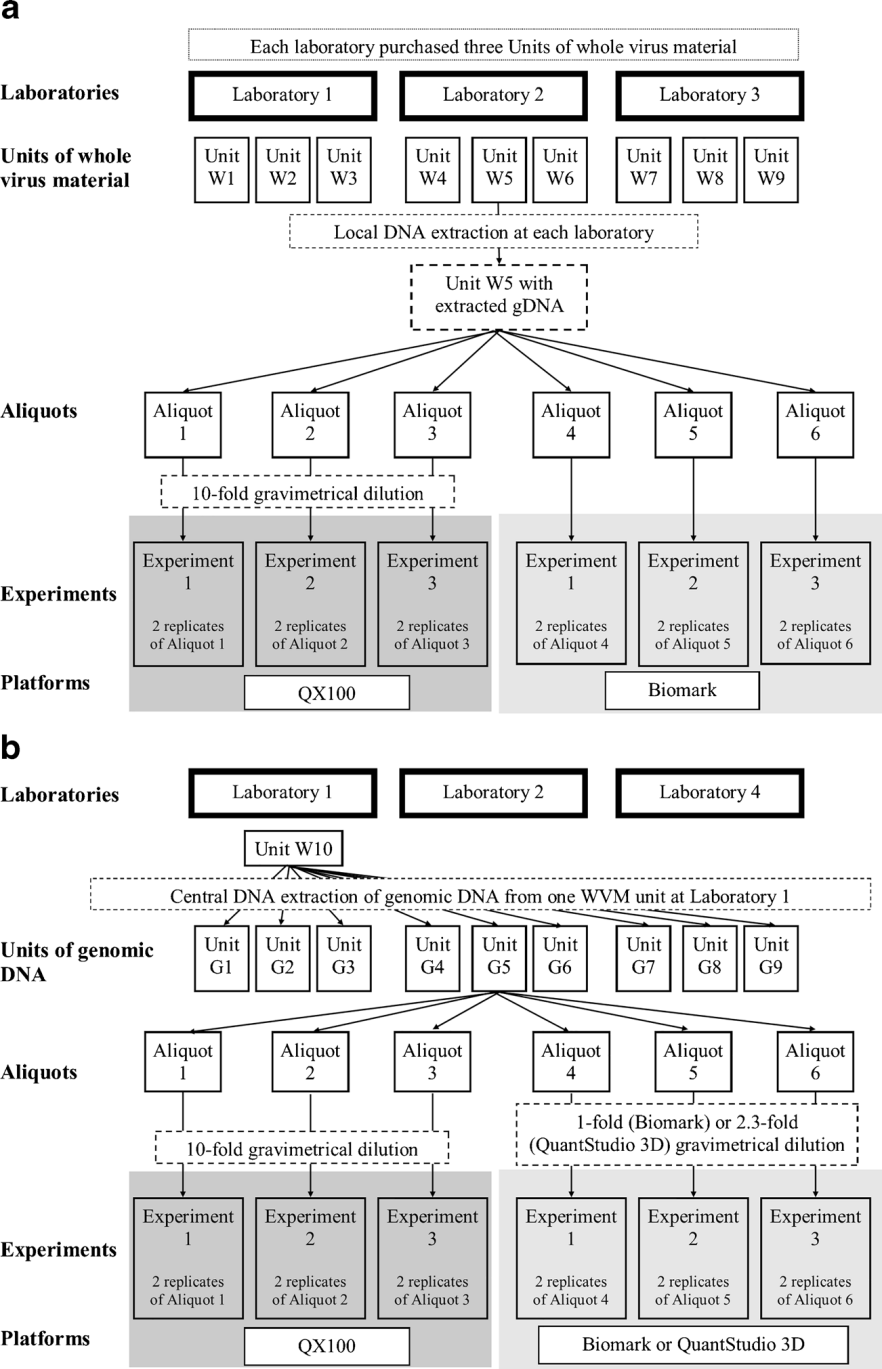
Schematical overview of the inter-laboratory assessment for **A** whole-virus material (WVM) and **B** genomic DNA (gDNA). With both HCMV test materials, the procedure presented for unit 5 was also applied to all of the other units (for WVM and gDNA; omitted here for clarity). For each HCMV test material within each laboratory, at least one of the two platforms shown was used

In the present study, inter-laboratory comparisons were conducted to determine the intermediate precision and reproducibility of different dPCR platforms for quantification of DNA extracted from a reference material from HCMV. Two HCMV test materials were used in four National Metrology Institutes (Fig. 1): whole-virus material (WVM), which was purchased from the manufacturer individually by each laboratory for subsequent ‘local’ DNA extraction (henceforth referred to as the ‘locally extracted WVM units’); and genomic DNA (gDNA), which was ‘centrally’ extracted from a single WVM unit in the National Institute of Biology (NIB) laboratory (Fig. 1, Table 1, laboratory 1), and aliquoted into the ‘gDNA units’ that were distributed to the collaborating laboratories (henceforth referred to as the ‘centrally prepared gDNA units’). In each participating laboratory, DNA quantification of the locally extracted WVM units and the centrally prepared gDNA units was performed using one or two of the following dPCR platforms: a droplet-based dPCR system (QX100 Droplet Digital PCR; Bio-Rad); and two chip-based dPCR systems (Biomark HD, Fluidigm; QuantStudio 3D Digital PCR, Thermo Fisher Scientific). Three units of each HCMV test material (as both the locally extracted WVM units and the centrally prepared gDNA units) were analysed on each dPCR instrument as three consecutive experiments, to determine the inter-unit variability and inter-experiment variability (i.e. intermediate precision, considering single unit measured in three experiments in duplicate) within each instrument. Additionally, to determine the reproducibility of the dPCR for each test material, and thus the potential for characterisation and value assignment of reference materials, the mean HCMV DNA copy number concentrations and corresponding measurement uncertainties were estimated using the data from all five dPCR instruments.

**Table 1:** Participating laboratories, dPCR platforms and HCMV test materials analysed in this study

Lab.	Institute	Country	dPCR platform	Manufacturer	Material analysed
1	NIB	Slovenia	QX100 Biomark	Bio-Rad Fluidigm	WVM, gDNA
2	Directorate F	European Union	QX100 Biomark	Bio-Rad Fluidigm	WVM, gDNA
3	LGC	UK	Biomark	Fluidigm	WVM
4	TUBITAK UME	Turkey	QuantStudio 3D	Thermo Fisher Scientific	gDNA

*NIB* National Institute of Biology; *Directorate F* Joint Research Centre, European Commission; *LGC* (formerly Laboratory of the Government Chemist); *TUBITAK UME* National Metrology Institute of Turkey; *WVM* whole-virus material; *gDNA* genomic DNA

## Materials and methods

### Test materials

#### Whole HCMV material for local DNA extraction

For the quantification of the locally extracted HCMV DNA from WVM, laboratory 1 obtained five units of the First World Health Organisation International Standard for Human Cytomegalovirus for Nucleic Acid Amplification Techniques (i.e. four WVM units) from the WHO (code, 09/162; UK), and each collaborating laboratory obtained four WVM units (Fig. 1, Table 1, laboratories 2–4). Each of these WVM units of the HCMV standard comprised the lyophilised equivalent of 1 mL of a whole-virus preparation of the HCMV ‘Merlin’ strain that was resuspended in 10 mM Tris-HCl buffer (pH 7.4) with 0.5% (*v*/*v*) human serum albumin. The material had been assigned a nominal HCMV concentration of 5 × 10^6^ IU/mL when reconstituted in 1 mL nuclease-free water, based on data from an international collaborative study. The uncertainty of the vial content determined by the manufacturer was ±0.23%. The material was shown to be stable during its shipment at ambient temperatures.

#### Centrally prepared HCMV genomic DNA

HCMV gDNA was centrally prepared at laboratory 1 (NIB; Fig. 1B, Table 1) by extraction of the gDNA from one WVM unit, followed by circulation of the prepared gDNA units to the participating laboratories. To prepare the gDNA units, one of WVM units (i.e., Fig. 1B, unit W10) obtained by laboratory 1 from WHO (code, 09/162; UK) was used. After opening of WVM unit, the contents were reconstituted in 1 mL double-distilled water, followed by fivefold dilution in phosphate-buffered saline (PBS) (137 mM NaCl, 2.7 mM KCl, 8 mM Na_2_HPO_4_, 2 mM KH_2_PO_4_, pH 7.4). This material was then divided into 22× 200-μL aliquots, from where the gDNA was extracted on the same day using QIAamp DNA Mini kits (Qiagen). These extractions were carried out according to the manufacturer instructions, except for the DNA elution, where only 50 μL elution buffer was used (instead of 200 μL). The extracted HCMV gDNA was then pooled, mixed and aliquoted into 50-μL aliquots (i.e. the gDNA units). The concentration of the HCMV gDNA was assigned during the homogeneity assessment, for which five of these gDNA units were tested (see Electronic Supplementary Material (ESM), Method S1, gDNA units H1–H5). The remaining gDNA units were stored at −20 °C for 4 months. Eight of these gDNA units were then sent on dry ice to two of the collaborating laboratories (Fig. 1, Table 1, laboratories 2, 4), as four gDNA units for each laboratory, while four of these gDNA units remained at laboratory 1.

### Participating laboratories and dPCR platforms

Four National Metrology Institutes participated in this collaborative study: laboratory 1, National Institute of Biology (NIB), Slovenia (the ‘central’ laboratory); laboratory 2, Joint Research Centre, European Commission, Directorate F. Retieseweg, European Union; laboratory 3, LGC (formerly Laboratory of the Government Chemist), United Kingdom; and laboratory 4, National Metrology Institute of Turkey (TUBITAK UME), Turkey. The dPCR platforms used and the materials tested (i.e., WVM units and gDNA units) by each of these participating laboratories are shown in Table 1.

### Experimental workflow

Three of the collaborating laboratories (Fig. 1, Table 1, laboratories 1–3) obtained at least four WVM units from the manufacturer and two participating laboratories (Fig. 1, Table 1, laboratories 2 and 4) also received four gDNA units from laboratory 1. One of each of these WVM units and gDNA units of the test materials was used for preliminary analysis of the analytical protocol, with the remaining three WVM units (e.g. Fig. 1A, units W1–W9) and gDNA units (e.g. Fig. 1B, units G1–G9) of each test material used in the collaborative study. Two vials with mixed primers and probes were also received by each collaborating laboratory (from laboratory 1) on dry ice (see below). The complete experimental workflow is shown schematically in Fig. 1. In each laboratory for each dPCR instrument, three WVM units and/or three gDNA units were included in this analysis. For each dPCR instrument, three experiments were performed on different days over a short period of time. In each experiment, three aliquots were tested simultaneously, with each derived from a different WVM or gDNA unit (e.g. Fig. 1B, experiment 1, aliquot 1 from unit G1, aliquot 1 from unit G2, aliquot 1 from unit G3). Laboratories 1 and 2 examined both test materials in the same experiment.

### DNA extraction of WVM units and aliquot preparation

In laboratory 1 and upon arrival at the collaborating laboratories (laboratories 2, 3), each WVM unit (e.g. Fig. 1A, units W1–W9) was opened and diluted in 1 mL double-distilled water, as stated by the manufacturer (final nominal HCMV concentration, 5 × 10^6^ IU/mL). At least 200 μL of this prepared material was additionally diluted fivefold in PBS (each collaborating laboratory used either the same PBS as for the laboratory 1 units, or purchased PBS from a manufacturer), to reach the nominal HCMV concentration of 1 × 10^6^ IU/mL. For each diluted material, DNA extraction was performed using High Pure Viral Nucleic Acid kits (Roche), according to the manufacturer instructions. Immediately after the elution of the DNA, each of three tubes with 50-μL eluted DNA was aliquoted as described below. Additionally, in each participating laboratory (laboratories 1, 2, 3), 200 μL negative extraction control (40 μL double-distilled diluted in 160 μL PBS) was extracted using High Pure Viral Nucleic Acid kits (Roche) simultaneously with the extraction of the WVM units. Following the extraction, the negative extraction control was aliquoted into nine 5 μL aliquots for subsequent analysis.

### Aliquot preparation and dilutions of test materials

In each laboratory, from each locally extracted gDNA (50-μL eluted volume) from each WVM unit, and from each centrally prepared 50-μL gDNA unit, six aliquots were prepared (e.g. Fig. 1A, aliquots 1–6 from unit W5), by dividing the 50-μL volume into six low-binding microcentrifuge tubes (aliquot volumes used for dPCR platforms: QX100, ∼6 μL; Biomark, ∼9 μL; QuantStudio 3D, ∼9 μL). All of the aliquots were then stored at −20 °C. For each dPCR instrument, three aliquots from the same WVM unit or gDNA unit were each analysed in one of three consecutive experiments on the same dPCR instrument, over a short period of time. Each aliquot was thawed, gravimetrically diluted in double-distilled water and analysed on the same day.

On the QX100 system, for quantification of the aliquots (derived from both the WVM units and the gDNA units), 10-fold gravimetric dilutions were made of each one on the day of the analysis. For the analysis on the Biomark system, undiluted aliquots derived from the WVM units and the gDNA units were used. In case of QuantStudio 3D system, 2.3-fold diluted aliquots of gDNA units were used. Each participating laboratory reported all of their gravimetric dilutions in Excel 2007 data submission spreadsheets (ESM, Tables S1-S6).

### PCR assay for dPCR

In all of the experiments, the same *UL54* assay was used for quantification of the HCMV DNA in the WVM units and the gDNA units, which targets the DNA polymerase (*UL54*) gene of HCMV [22] (ESM, Table S7). The *UL54* assay had been assessed previously for its robustness on the QX100 system and the Biomark system, with well-defined dynamic ranges and limits of quantification and detection obtained [23]. Laboratory 1 prepared 20-fold concentrated mixtures of 600 nM oligonucleotide primers, and 200 nM probes which were mixed, aliquoted (75-μL aliquots) and stored at −20 °C. Two aliquots were then shipped to each of the collaborating laboratories (laboratories 2–4) on dry ice, where they were stored at −20 °C.

### dPCR

Three different dPCR platforms were used in this study (Table 1). Two laboratories used a droplet-based dPCR platform (laboratories 1, 2; QX100 system, Bio-Rad), three laboratories used a chip-based dPCR platform from Fluidigm (laboratories 1–3; qdPCR 37K Integrated Fluidic Circuits for the Biomark system; henceforth referred to as the Biomark 37K array), and one laboratory used a chip-based dPCR platform from Thermo Fisher Scientific (laboratory 4; QuantStudio 3D system). For each instrument, the experiments were performed according to the guidelines from the minimum information for publication of quantitative digital PCR experiments (ESM, Table S8).

For the Biomark 37K array, 8-μL reactions with excess volume were used, which comprised 2 μL 4× TaqMan Fast Virus 1-Step Master Mix (Thermo Fisher Scientific); 0.4 μL 20× *UL54* assay; 0.8 μL GE Sample Loading Reagent (Fluidigm); 2 μL double-distilled water; and 2.8 μL sample. In each experiment, three no template controls (NTCs) and three aliquots of the negative extraction control were included. As only 24 samples were pipetted into the 48-inlet arrays, the remaining 24 inlets were filled with no template reaction mix, to avoid baseline problems (5-μL reactions with excess volume composed of 1.25 μL 4× TaqMan Fast Virus 1-Step Master Mix, 0.5 μL GE Sample Loading Reagent, and 3.25 μL double-distilled water). During the array loading, only 4-μL reactions were loaded into the 770 chambers of each inlet, while the excess reaction volume served to reduce the bias that can arise from small pipetting volumes. The reactions were performed using universal conditions: 2 min at 50 °C, 10 min at 95 °C, followed by 45 cycles of 15 s at 95 °C and 1 min at 60 °C. Ramp rate was set to 2 °C/s. The analyses were performed using different versions of Biomark HD Data Collection Software (Fluidigm). Each participating laboratory reported the version of the analysis software, the fluorescence threshold, the quality threshold, the accepted Cq range, the baseline correction method and the number of positive amplifications per panel, with all included in their data submission Excel 2007 spreadsheets (ESM, Table S9).

For the QX100 system, 20-μL reactions were used, composed of 10 μL 2× ddPCR Supermix for Probes (Bio-Rad Laboratories, USA); 1 μL 20× *UL54* assay; 1 μL double-distilled water; and 8 μL sample. Three NTCs were included in each experiment. The reactions were performed using the same universal conditions as for the Biomark system, except for the addition of a single incubation of 10 min at 98 °C at the end of the cycling. Each participating laboratory reported the version of QuantaSoft analysis software used (Bio-Rad), the method for determination of the fluorescence threshold (manual or automatic), the fluorescence threshold, the number of accepted droplets and the number of positive droplets, with all included in their data submission Excel 2007 spreadsheets (ESM, Table S10). Additionally, each laboratory reported 2D charts of each experiment to allow exclusion of measurements with abnormally increased droplet fluorescence [14] (ESM, Fig. S1).

For the QuantStudio 3D (TUBITAK UME only), 15-μL reactions were used, composed of 7.5 μL 2× QuantStudio 3D Digital PCR Master Mix kits (Thermo Fisher Scientific.); 0.75 μL 20× *UL54* assay; and 6.75 μL sample. These were loaded into the QuantStudio 3D Digital Chips (version 1). Two NTCs were included in each experiment. The reactions were carried out under the following cycling conditions: 10 s at 96 °C, 39 cycles of 60 s at 60 °C and 30 s at 96 °C and 10 min at 60 °C. For each chip/reaction, three readings were used and the means of the three readings are given in ESM, Table S6. The analyses were performed with QuantStudio 3D AnalysisSuite Software version 1.1.1 (Thermo Fisher Scientific), the quality threshold was set to 0.5 in the ‘colour by quality’ mode and an automatically calculated threshold was used in the ‘colour by calls’ mode to separate the positive and negative signals. The number of negative chambers and the number of qualified chambers after application of the quality threshold setting were reported, with all included in the data submission Excel 2007 spreadsheets. A more recent update from manufacturer on the partition volume (0.809 nL) was used for estimation of the DNA copy number concentration, instead of the partition volume of 0.865 nL that was the announced chip-partition volume of the first version of the QuantStudio 3D Chip kit.

### Analysis of results

#### Calculation of DNA copy number concentration

For each of the laboratories and dPCR instruments, the estimated DNA copy number concentration (cp/μL) for each of the aliquots derived from the WVM units and the gDNA units were based on the reported dilutions, numbers of positive partitions and analysed partitions. For the QX100 system, the droplet volume was determined by Corbisier et al. to be 0.834 nL [14], with the QuantStudio 3D, the updated chamber volume of 0.809 nL was used, while the partition volumes of the Biomark 37K array were defined by the manufacturer (ESM, Table S11). The equations used to calculate the initial DNA copy number concentration were reported in our previous study [23]. For each aliquot, the estimated DNA copy number concentration was calculated according to the means of two replicates analysed in a single experiment.

#### Statistical analysis

The homogeneity and stability of the gDNA units were determined using single-factor analysis of variance (ANOVA) in R studio, version 0.98.977. For the homogeneity study, the mean DNA copy number concentrations for each of the five gDNA units were measured (ESM, Method S1, gDNA units H1-H5), with duplicate measurements for each gDNA unit taken into account. For the stability study, six replicates were taken into account for each of three gDNA units (ESM, Method S1, gDNA units G1-G3) that were also tested as a part of the inter-laboratory study. For the inter-laboratory study and stability study, outliers were determined for each instrument independently, using Grubbs tests (R studio, package ‘outliers’), and these were excluded from the further analysis. For the intra-experiment variability, the coefficients of variation (CVs) were calculated for the duplicates in Excel 2007, by dividing the standard deviation by the mean DNA copy number concentration. With the intermediate precision, CVs were calculated from all measurements performed in three experiments on the same instrument using one gDNA or WVM unit (*n* = 6). Statistical analyses of inter-unit variability within a single instrument, and those that assessed the reproducibility between the instruments and laboratories, were performed using ANOVA and Tukey’s tests (R studio). For each instrument and HCMV test material, the estimated mean DNA copy number concentrations were calculated by taking the means of all of the data from every unit (WVM units or gDNA units) and experiment (Microsoft Excel 2007). To evaluate the differences between the reported mean DNA copy number concentrations from the different dPCR instruments, ANOVA and Tukey’s tests were used, in R studio. In addition, the standard measurement uncertainties were calculated for every instrument and HCMV test material, based on the bottom-up approach in Eq. (1) [24]:

(i) The measurement uncertainty was calculated as a combined measurement uncertainty (*MU*) according to Eq. (1):
  1 $$ M U=\sqrt{{u_r}^2 + {u_{ip}}^2\ } $$
  where *u*_*r*_ is the uncertainty associated with the repeatability, and *u*_*ip*_ is the uncertainty associated with the intermediate precision.
(ii) *u*_*r*_ and *u*_*ip*_ were calculated using Eqs. (2), (3) and (4):
  2 $$ {u}_r=\frac{\sqrt{M{ S}_{\mathrm{within}}}\ }{\sqrt{n}} $$
  3 $$ {u}_{ip1}=\sqrt{\frac{M{S}_{\mathrm{between}} - M{S}_{\mathrm{within}}}{n \times N}} $$
  4 $$ {u}_{ip2}=\frac{\sqrt{\frac{M{ S}_{\mathrm{between}}\ }{n}} \times \sqrt[4]{\frac{2}{N\times \left( n-1\right)}}}{\sqrt{N}} $$
  where *n* is the number of independent replicates per experiment, *N* is the number of experiments performed on one instrument, *MS*_within_ is the mean square value within groups and *MS*_between_ is the mean square value between groups. Both mean squares were calculated using ANOVA in Microsoft Excel 2007, with all of the measurements taken into account for each experiment, including duplicates. If *MS*_between_ > *MS*_within_, Eq. (3) was used to calculate the intermediate precision. In contrast, if *MS*_between_ < *MS*_within_, Eq. (4) was used to calculate the intermediate precision. To obtain the expanded measurement uncertainty, the coverage factor (*k*) was applied. The value of the coverage factor was chosen at the 95% level of confidence, based on the degrees of freedom (i.e., *k* = 2.2 for two experiments, each with six independent replicates [*n* = 12]; *k* = 2.11 for three experiments, each with six independent replicates [*n* = 18]).

For both of the HCMV test materials, the mean DNA copy number concentrations and the corresponding expanded measurement uncertainties, which took into account the mean DNA copy number concentration of each instrument, were estimated according to the guidelines from the Consultative Committee for Amount of Substance: Metrology in Chemistry and Biology (CCQM) [25]. To select the most appropriate estimator, the following were performed: Grubbs test for outliers (R studio); preliminary graphical inspection to check for over-dispersion of mean values; mutual consistency check (chi-squared test in Excel 2007) [25]; and Birge ratio calculation (ESM, Method S2). For both of the HCMV test materials, estimation of the mean DNA copy number concentration and the corresponding measurement was performed using the Vangel-Ruhkin estimator (R studio; package ‘metrology’), which was characterised as the most appropriate estimation method based on the CCQM guidelines (ESM, Method S3) [25].

## Results and discussion

### Central laboratory monitoring of concentration, homogeneity and stability of gDNA

To assign nominal DNA copy number concentration to the gDNA material and to define the homogeneity of the gDNA units, initial monitoring was carried out in laboratory 1. Here, five gDNA units (units H1–H5) were analysed in duplicate on a QX100 system immediately after the gDNA extraction from a single WVM unit (unit W10). The stability of the test batch was checked during the inter-laboratory study with the analysis of three gDNA units (units G1–G3) that had been stored for 4 months at −20 °C. The mean DNA copy number concentration of the gDNA test material (combined DNA concentrations from units H1–H5 and G1–G3) (±expanded standard error; *k* = 2.78) was estimated at 979 (±59) cp/μL (ESM, Fig. S2), and the gDNA units H1–H5 were homogenous in terms of the DNA copy number concentration (*p* > 0.41; ANOVA 95% confidence level). By comparing units G1–G3 with units H1–H5, the stability of the gDNA units after the 4 months of storage at −20 °C was also confirmed (*p* > 0.9; ANOVA 95% confidence interval) (ESM, Fig. S2).

### Inter-laboratory assessment

The two different HCMV test materials comprised the locally extracted gDNA from the purchased WVM units (e.g. Fig. 1A, units W1–W9), and the centrally prepared gDNA units (e.g. Fig. 1B, units G1–G9) that were distributed to the participating laboratories. These were each quantified in three different laboratories using two or three different dPCR platforms (Table 1). In each laboratory, three WVM units and three gDNA units were tested. From each WVM unit and gDNA unit, six aliquots were locally prepared, three aliquots for subsequent analysis on one dPCR platform (laboratories 1–4) and three aliquots for analysis on another dPCR platform (laboratories 1, 2). For each dPCR instrument, three aliquots derived from each of the three WVM units and three gDNA units were each tested in duplicate in one of three consecutive experiments (e.g. Fig. 1A, aliquots 1–3 from unit W5 were tested in experiments 1–3), to determine the intermediate precision and the inter-unit variability within each dPCR instrument and laboratory. Additionally, for each dPCR instrument, the mean DNA copy number concentration and the corresponding expanded measurement uncertainty were estimated. For each of the three WVM units and three gDNA units, three of the four participating laboratories reported complete data for the three units tested in three experiments on one or two dPCR platforms (ESM, Tables S1-S6). The exception here was laboratory 1, which reported only two complete sets of data due to technical problems with the Biomark system (ESM, Table S1). With all of the instruments, the NTCs and negative extraction controls were negative, except for the QuantStudio3D system, where one false positive partition was noted in two NTCs. This can occur due to cross-contamination between samples or because of non-specific binding of primers [13]. However, due to the high DNA copy number concentrations used in this inter-laboratory study, the occurrence of a single false positive partition in the NTCs did not bias the subsequent interpretation of the data.

### Intra-experiment variability, intermediate precision and agreement between experiments

For each dPCR instrument, three experiments were performed, each with one of three aliquots derived from each of three WVM units and three gDNA units, analysed in duplicates. Grubbs tests were used to determine the outliers and exclude these from the further analysis (ESM, Table S12). With each of these HCMV test materials, low CVs related to intra-experiment variability were observed among the different dPCR instruments, as the great majority of the duplicates (e.g. Fig. 1A, two replicates of aliquot 1 from unit W5) had CV <10%, with some CVs between 10 and 40% mostly for the Biomark system. The low CVs related to the intra-experiment variability of the QX100 system and the Biomark 37K array are in agreement with other reports using HCMV DNA and bacterial DNA [26, 27]. The higher CVs observed with the Biomark 37K array compared to the other two dPCR platforms might have been due to the >25-fold smaller number of analysed partitions, and/or to pipetting errors related to the smaller sample volumes [23, 27].

**Fig. 2:**
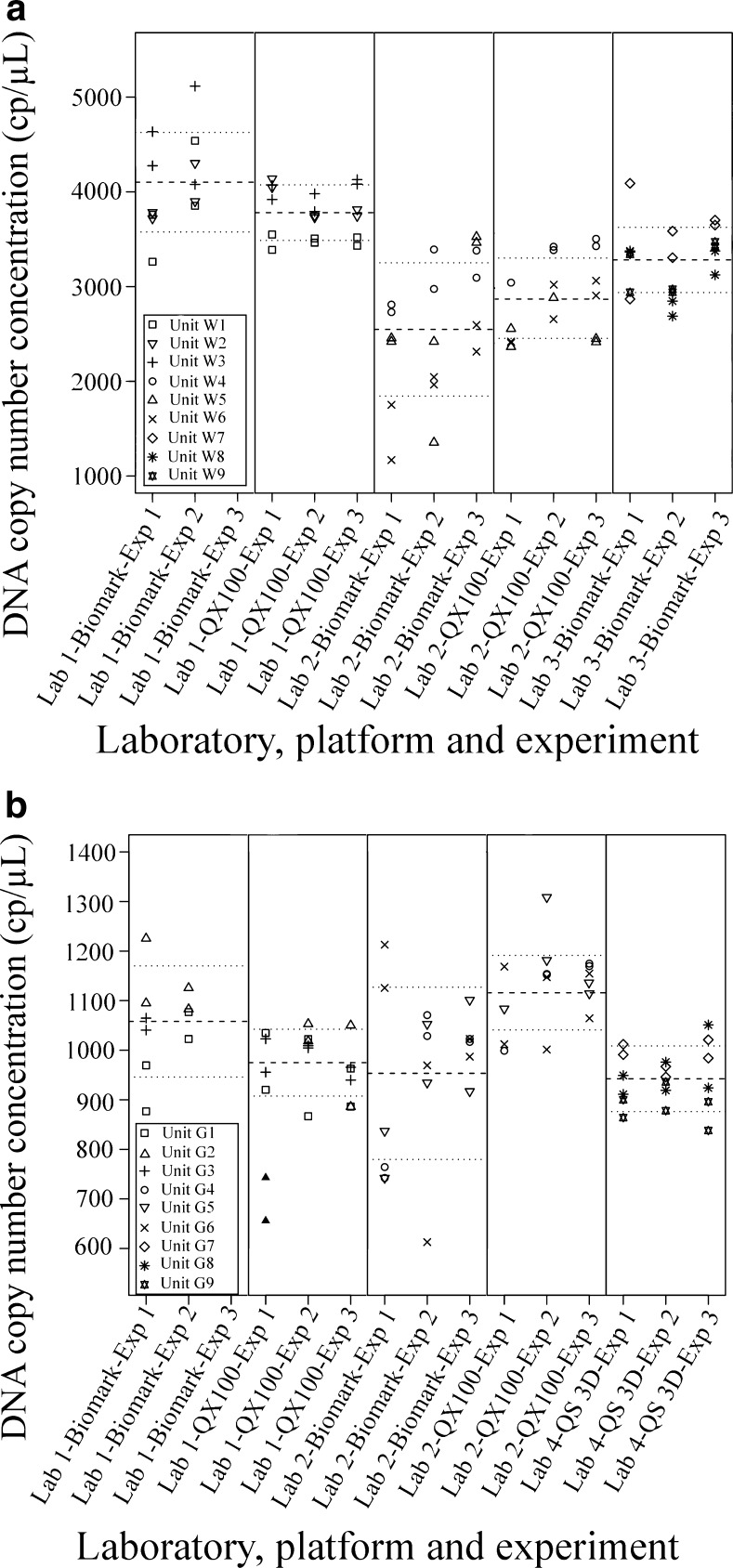
Mean DNA copy number concentration estimated on each of dPCR instrument involved in quantification of **A** whole-virus material (WVM) and **B** genomic DNA (gDNA). Each *symbol* denotes a single measurement of the dPCR platform, whereas *black triangles* represent outliers. *Dashed lines*, mean DNA copy number concentration obtained on each dPCR instrument in all experiments; *dotted lines*, expanded measurement uncertainty considering all experiments (Lab 1-Biomark, *k* = 2.2; all other instruments, *k* = 2.11)

With all of the dPCR instruments, low CVs related to intermediate precision were noted (CVs below 25%). Moreover, with each HCMV test material tested with each dPCR instrument, there were no statistically significant differences in the mean DNA copy number concentration between the three consecutive experiments, when for each all six measurements (three units in duplicate) were taken into account (Fig. 2, ESM, Table S13). The low CVs related to intermediate precision are in agreement with previous reports from individual laboratory data for HCMV DNA and different bacterial DNA template types [26, 28]. This finding provides additional support for the indications that dPCR is suitable as a reference measurement procedure, as it provides very precise quantification of viral DNA within and between experiments.

#### Inter-unit variability

With the gDNA test material, each of the four participating laboratories measured three different gDNA units that were centrally prepared in laboratory 1. In contrast, for the WVM test material, the DNA from three WVM units was locally extracted and analysed in each of the three laboratories. With all of the dPCR instruments, the centrally prepared gDNA units showed only minor inter-unit variability, as the differences in mean DNA copy number concentration between these gDNA units were below 14% and were mostly not statistically significant (Table 2; ESM, Fig. S3). On the other hand, for the WVM units, where the gDNA was extracted locally, there was higher inter-unit variability compared to the centrally prepared gDNA units, with the highest difference between the WVM units with the Biomark 37 array from laboratory 2, with 55% higher mean DNA copy number concentration obtained from unit W4 compared to unit W6 (Table 2; ESM, Fig. S4). The low CVs related to inter-unit variability of the centrally prepared gDNA units confirms their homogeneity when analysed in each of the participating laboratories. This is in agreement with other reports where simple DNA templates that did not require DNA extraction were used (e.g. gDNA, plasmid DNA) [28, 29]. Despite the statistically significant differences between the centrally prepared gDNA units tested on the QuantStudio 3D system, there were low CVs related to intra-experiment variability (CVs for duplicates, <10%) and intermediate precision (CVs for three experiments, <1%), which explains the statistical significance of <12% difference between these gDNA units. With the WVM material tested in laboratories 1 and 2, most of the differences between the three analysed WVM units were constant, as they were observed with both dPCR platforms. With the low filling-volume related uncertainty and high stability of the WVM units that are claimed by the manufacturer, it is reasonable to assume that the inter-unit variability was introduced during the DNA extraction, as the DNA was locally extracted from each individual WVM unit. This is in agreement with previous studies where up to 50% difference was noted between DNA-extraction replicates quantified using the same qPCR assay [30, 31]. With PCR-based DNA quantification, estimation of DNA copy number concentration can be influenced by variable DNA recovery and/or insufficient removal of PCR inhibitors during DNA extractions [32]. However, as dPCR platforms are considered to be relatively robust to potential inhibitory substances that might have remained during the DNA extraction [11, 12], it is likely that the differences in the estimated mean DNA copy number concentration between the WVM units were mostly caused by variable DNA recovery upon extraction. This is in agreement with previously reported data with HCMV, where intermediate variability was noted between extraction replicates analysed by dPCR within a single laboratory [20, 31]. With the QX100 system, the differences between the WVM units had higher statistical significance in comparison to the Biomark 37K array. This finding suggests that the QX100 system provides more precise discrimination between the WVM units. This is due to the lower CVs related to the intra-experiment variability of the QX100 system compared with the Biomark 37K array, as previously discussed.

**Table 2:** Inter-unit variability and intermediate precision obtained on different platforms and instruments. For every unit in each laboratory, mean DNA copy number concentrations were calculated considering all duplicate measurements from all experiments (*n* = 6 or less). *p* value denotes statistical significance of differences between units. For each unit from every laboratory, intermediate precision is calculated as CV considering all measurements performed in three experiments (*n* = 6 or less)

HCMV test material	Lab.	Platform	First unit^a^		Second unit^b^		Third unit^c^		ANOVA
			Mean DNA copy number (cp/μL)	CV (%)	Mean DNA copy number (cp/μL)	CV (%)	Mean DNA copy number (cp/μL)	CV (%)	*p* value
WVM	1	Biomark	3853	14	3926	7	4526	10	0.10
WVM	1	QX100	3476	2	3870	5	3995	3	<0.001
WVM	2	Biomark	3063	9	2606	31	1973	25	0.02
WVM	2	QX100	3355	5	2532	8	2741	11	<0.001
WVM	3	Biomark	3533	12	3129	10	3182	8	0.10
gDNA	1	Biomark	986	9	1132	6	1053	2	0.060
gDNA	1	QX100	949	7	1002	8	983	3	0.40
gDNA	2	Biomark	941	14	931	13	988	19	0.81
gDNA	2	QX100	1130	7	1128	4	1091	7	0.26
gDNA	4	QuantStudio 3D	987	3	955	6	885	4	0.002

*WVM* whole-virus material, *gDNA* genomic DNA

^a^First unit, WVM: units W1 (laboratory 1), W4 (laboratory 2), W7 (laboratory 3); gDNA: units G1 (laboratory 1), G4 (laboratory 2), G7 (laboratory 4)

^b^Second unit, WVM: units W2 (laboratory 1), W5 (laboratory 2), W8 (laboratory 3); gDNA: units G2 (laboratory 1), G5 (laboratory 2), G8 (laboratory 4)

^c^Third unit, WVM: units W3 (laboratory 1), W6 (laboratory 2), W9 (laboratory 3). gDNA: units G3 (laboratory 1), G6 (laboratory 2), G9 (laboratory 4)

#### Measurement uncertainties of each dPCR instrument

With each HCMV test material and dPCR instrument, the mean DNA copy number concentrations and corresponding expanded measurement uncertainties were calculated by taking into account the three WVM units or gDNA units, each of which was divided into three aliquots that were each measured in one of three experiments. For every dPCR instrument, small expanded measurement uncertainties (<18%) were obtained for the centrally prepared gDNA test material, whereas with a more complex material (i.e. WVM) that requires local DNA extraction, higher expanded measurement uncertainties (<28%) were noted (Fig. 2, Table 3). This is in agreement with a previous inter-laboratory study on bacterial DNA [28]. Additionally, in two other assessments using simple and well-defined DNA templates [29, 33], lower expanded measurement uncertainties (<6%) were observed for the QX200 system, the Biomark 12.765 arrays and other dPCR platforms compared to this study. Hence, dPCR offers a very precise estimation of the DNA copy number concentration. However, the final measurement uncertainty is dependent on the complexity of the DNA material, with the local DNA extraction resulting in additional uncertainty components, i.e., leading to higher measurement uncertainty. In the present study, within laboratories 1 and 2, smaller measurement uncertainties were seen for the QX100 system compared to the Biomark 37K array (Fig. 2, Table 3), which is in agreement with another report where a QX100 system and a Biomark 12.765 array were compared [33]. This might arise from the higher CVs related to the intra-experiment variability that was noted on the Biomark 37K system. As no such differences between the QX100 and the Biomark 37K arrays were observed in the inter-laboratory study on bacterial DNA, this might be attributed to the study setup and pipetting errors [28].

**Table 3:** Estimated mean DNA copy number concentrations and expanded measurement uncertainties

Lab.	Platform	Whole-virus material		Genomic DNA material	
		Mean DNA copy number (cp/μL)	Relative expanded measurement uncertainty (%)^a^	Mean DNA copy number (cp/μL)	Relative expanded measurement uncertainty (%)^a^
1	Biomark	4101	13	1058	11
1	QX100	3780	8	974	7
2	Biomark	2547	28	953	18
2	QX100	2867	15	1114	6
3	Biomark	3281	10	/	/
4	QuantStudio 3D	/	/	942	7

^a^Biomark from laboratory 1, *k* = 2.20; all other instruments, *k* = 2.11

#### Intra-laboratory agreement

In laboratories 1 and 2, two different dPCR platforms were used. For both HCMV test materials analysed on the Biomark 37K array in laboratory 1, the mean DNA copy number concentration was approximately 8% higher than that measured on the QX100 system (Table 3). The opposite was noted in laboratory 2, where both of the HCMV test materials showed 17% lower mean DNA copy number concentrations when measured on the Biomark 37K array compared with those measured using the QX100 system. The high intra-laboratory agreement between the QX100 system and the Biomark 37K array has already been observed in two other studies [23, 27]. Additionally, low discrepancies were observed between the other dPCR platforms [14, 20, 33].

Within laboratories 1 and 2, the differences between the QX100 system and the Biomark 37K array were very consistent, as the differences in the DNA copy number concentration between both of these platforms were similar, regardless of the HCMV test material used. Similar consistency between these two platforms has already been noted for three different types of bacterial DNA [28].

However, there was disagreement noted between laboratories 1 and 2, where for the QX100 system, higher (laboratory 2) and lower (laboratory 1) mean DNA copy number concentrations were measured compared to the Biomark 37K array. Although in both laboratories differences between those two platforms were smaller than expanded measurement uncertainty of each platform, this pattern was noted with both test materials. Similar inconsistent data have been reported previously for plasmid DNA [28], which suggests that such discrepancies between platforms are not always systematic, but can be random; however, the reasons for such random discrepancies are not yet completely understood. As the same assay, and the same HCMV test materials and cycling conditions were used in all of the laboratories, over-estimation and under-estimation of the DNA copy number concentrations and discrepancies between laboratories might be due to either the use of different master mixes, or to incorrectly assigned partition volumes [15, 23, 27]. For both dPCR platforms, various lot numbers of the particular master mixes were used in the different laboratories, which might partially contribute to these observed discrepancies between the platforms. With the QX100 system and the Biomark 12.765 array, >10% difference in partition volume was reported from an independent assessment carried out in several laboratories [14, 33–35]. Furthermore, with the Biomark 12.765 array, around a 7% difference in chamber volume was found between two arrays measured in the same laboratory [36]. The discrepancy between the QX100 system and the Biomark 37K array observed in the present study might therefore arise from variable partition volumes of the different Biomark 37K array lots and/or differences between droplet volumes generated and analysed for the QX100 systems from different laboratories.

#### Inter-laboratory agreement and mean DNA copy number concentrations

The mean DNA copy number concentrations for each HCMV test material were measured on each dPCR instrument (i.e. two QX100 systems, two Biomark systems, one QuantStudio 3D) from the four laboratories. With the gDNA test material, the differences between the laboratories did not exceed the differences within each laboratory, as the maximum difference in mean DNA copy number concentration between the dPCR instruments from two laboratories was <20% (Table 3). Furthermore, no statistically significant differences were observed between the majority of the instrument pairs (ESM, Fig. S5A). Between laboratories 1 and 2, there was only a minor difference in mean DNA copy number concentrations when the mean DNA copy number concentrations from the dPCR platforms within each laboratory were taken into account. Conversely, with the more complex DNA material of WVM, a 62% difference in the mean DNA copy number concentration was noted between the two Biomark instruments from different laboratories (Table 3), with statistically significant differences between most of these instrument pairs (ESM, Fig. S5B). Furthermore, in laboratory 1, approximately 40% higher mean DNA copy number concentrations were noted when compared to laboratory 2.

With the gDNA test material, the good agreement between the laboratories additionally demonstrated the high stability of the gDNA units distributed to the participating laboratories. The reasons for minor discrepancies between instruments and laboratories are still not well understood; however, they were probably primarily caused by each individual dPCR instrument, due to over-estimation or under-estimation of DNA copy number concentration, and are not directly influenced by factors related to the different laboratories. In contrast to the centrally prepared gDNA test material, the WVM required local DNA extraction before DNA quantification. DNA extraction has already been demonstrated to introduce an additional variability (CV up to 50%) due to differences in DNA recoveries from extraction columns of the same manual extraction kit used by one operator within one laboratory [20, 30, 31]. To the best of our knowledge, no inter-laboratory assessment of the same DNA extraction method for quantification of viral DNA has been performed. However, it can be speculated that different operators from different laboratories would contribute to this variability. Another source of variability might be differences in composition between the suggested in-house prepared PBS (laboratory 1) and the purchased commercial PBS (laboratories 2–4), as it has been shown that the matrix can have an impact on the DNA recovery and the variability of DNA extractions [20]. Therefore, it is reasonable to assume that in the present study, the local DNA extractions from WVM performed individually in each laboratory contributed to the higher discordance between the laboratories and instruments than for those observed with the centrally prepared gDNA.

**Fig. 3:**
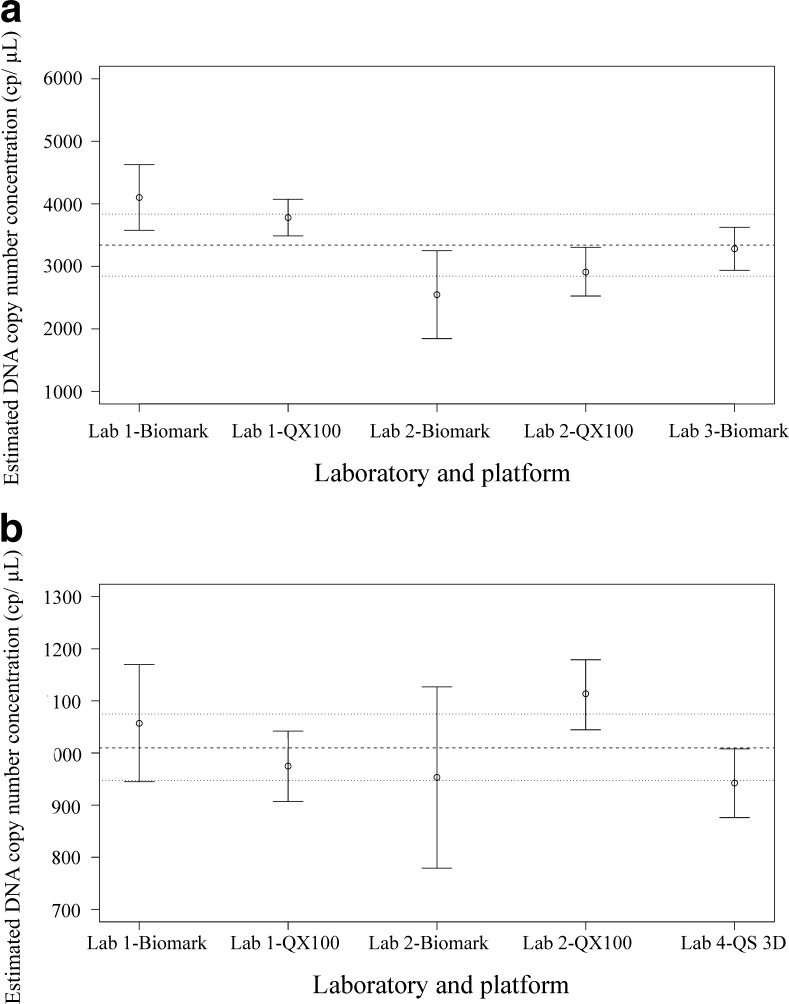
Estimated mean DNA copy number concentration and corresponding expanded measurement uncertainty for **A** whole-virus material (WVM) and **B** genomic DNA (gDNA). Each *dot* represents the mean DNA copy number concentration measured with one dPCR instrument, while *vertical bars* depict their corresponding expanded measurement uncertainty considering all material units tested in all experiments (Lab 1-Biomark, *k* = 2.2; all other instruments, *k* = 2.11). *Dashed line*, estimated mean DNA copy number concentration according to Vangel-Ruhkin estimator; *two dotted lines*, corresponding expanded measurement uncertainty

To additionally demonstrate the suitability of dPCR as a candidate reference measurement procedure of higher metrological order, its applicability for value assignments of different virus reference materials was determined. For each material, the Vangel-Ruhkin estimator was selected based on several criteria published in the CCQM guidelines [25] (ESM, Method S3, Tables S14, S15). With both materials, the data from all five of the dPCR instruments fell within narrow expanded measurement uncertainties (WVM, 15%; gDNA, 6%) of the mean DNA copy number concentrations (Fig. 3).

As the low measurement uncertainties observed in the present study are in agreement with two other inter-laboratory assessments that used bacteriophage DNA and different types of bacterial DNA [28, 29], we can conclude that dPCR offers good reproducibility for quantification of DNA of different complexities (e.g. plasmid DNA, gDNA, whole bacteria and viruses) and from different sources (e.g. viruses, bacteria, bacteriophages). To determine the suitability of dPCR as a reference measurement procedure and for characterisation of certified reference materials, the performance of dPCR should be compared to that of the qPCR method that is currently used for characterisation of virus reference materials [8]. The use of qPCR in several inter-laboratory studies for the quantification of HCMV resulted in more than 100-fold differences between laboratories in terms of the DNA copy number concentration [2, 8]. The main reason for this variability is most probably the disparity in the quantification procedures between the participating laboratories, as different assays and DNA extraction methods, and variable calibration procedures, can exacerbate the agreement between laboratories in terms of estimated DNA copy number concentration. In contrast to qPCR, several dPCR platforms have already been shown to be resilient to inhibitors and resistant to the influence of different PCR components, hence allowing for more accurate and robust quantification of DNA than is possible with qPCR [11, 23, 27]. The variability caused by the DNA extraction method in particular should receive special attention when dPCR is considered as a candidate for a reference measurement procedure and for characterisation of reference materials. The accuracy and robustness of dPCR-based DNA quantification can be further improved by preliminary selection of the DNA extraction method with the highest DNA recovery, while extraction replicates would probably reduce the influence of inter-column variability. Moreover, DNA recovery of the selected extraction method should be assessed to allow the inclusion of extraction related variability into the measurement uncertainty of quantification of the whole-virus reference material. Furthermore, where possible, dPCR-based direct quantification can bypass most of the mentioned problems, including inter-laboratory variability caused by the different operators of the extraction and clean up procedures, as this does not require DNA extraction and has been demonstrated to provide accurate and repeatable quantification of DNA derived from different whole-virus reference materials [20].

## Conclusions

To the best of our knowledge, the present study represents the first inter-laboratory assessment of different dPCR platforms for quantification of viral DNA. Two HCMV test materials, as WVM for local extraction and centrally prepared (extracted) gDNA, were selected to determine the repeatability, intermediate precision, and agreement in the quantification within and between laboratories (reproducibility) when using different dPCR platforms and instruments. For each dPCR instrument, good precision was seen. Furthermore, less than twofold differences in the estimated mean DNA copy number concentration were observed between dPCR platforms from different laboratories. As a consequence, when measurements from all participating dPCR instruments were considered, with both of these HCMV test materials, the mean DNA copy number concentrations were estimated with small expanded measurement uncertainties. All of these findings indicate that dPCR offers very repeatable (i.e. within instrument) and reproducible (i.e. between instruments, platforms and laboratories) quantification of viral DNA. This demonstrates that dPCR has the potential for implementation in the metrological framework as a measurement reference procedure, if correctly validated DNA extraction and quantification can be assured. In their current format, such methods could enable reproducible production of reference materials. They could also be applied to value assign secondary reference materials for calibration of the qPCR methods that are widely used.

## Electronic supplementary material

Below is the link to the electronic supplementary material.

ESM 1 (PDF 1220 kb)

## Abbreviations

37K array: qdPCR 37K integrated fluidic circuits for the Biomark system
ANOVA: Analysis of variance
CV: Coefficient of variation
dPCR: Digital polymerase chain reaction
gDNA: Genomic DNA
HCMV: Human cytomegalovirus
IU: International units
NIST: National Institute of Standards and Technology
NTC: No template control
PBS: Phosphate-buffered saline
PCR: Polymerase chain reaction
qPCR: Quantitative polymerase chain reaction
*UL54*: DNA polymerase gene of human cytomegalovirus
WHO: World Health Organisation
WVM: Whole-virus material
