# Supplementary material for: Inter-laboratory assessment of different digital PCR platforms for quantification of human cytomegalovirus DNA
Source: Anal Bioanal Chem. 2017 Jan 26;409(10):2601–14. doi: 10.1007/s00216-017-0206-0 (PMC5359388; doi:10.1007/s00216-017-0206-0)
Supplement: Supplementary file 1 — (PDF 1220 kb) [file 216_2017_206_MOESM1_ESM.pdf]

Analytical and Bioanalytical Chemistry

Electronic Supplementary Material

Inter-laboratory assessment of different digital PCR platforms for quantification of human cytomegalovirus DNA

Jernej Pavšič, Alison Devonshire, Andrej Blejec, Carole A Foy, Fran Van Heuverswyn, Gerwyn M Jones, Heinz Schimmel, Jana Žel, Jim F Huggett, Nicholas Redshaw, Maria Karczmarczyk, Erkan Mozioğlu, Sema Akyürek, Müslüm Akgöz, Mojca Milavec

Method S1 Characterisation of gDNA and determination of its homogeneity and stability.

For the homogeneity study, five gDNA units (units H1–H5) from the same test sample batch were analysed in duplicate on the QX100 system in Laboratory 1, using the same protocol as given in the Material and methods section. As well as the homogeneity assessment, the measured values were also used to estimate the mean DNA copy number concentration of the prepared gDNA test material. This was calculated based on the means of all five of the gDNA units. Additionally, due to a shortage in the number of units rqeuired, the stability of the gDNA units was tested during the inter-laboratory assessment, with three gDNA units (units G1-G3) analysed on the identical QX100 system after four months of storage at -20 °C.

Method S2 Calculation of the Birge ratio, to assess the statistical consistency of the comparison.

To calculate the Birge ratio, the reference value h_{ref} and the corresponding combined standard uncertainty $u_c(h_{ref})$ need to be calculated, as:

$$h_{ref} = \frac{\sum_{i=1}^{n} u^{-2}(h_i) h_i}{\sum_{i=1}^{n} u^{-2}(h_i)} \quad (1),$$

$$u_c(h_{ref}) = \sqrt[-2]{\left(\sum_{i=1}^{n} u^{-2}(h_i)\right)} \quad (2),$$

where h_i is the reported mean DNA copy number concentration and $u(h_i)$ is the reported standard measurement uncertainty.

The Birge ratio (R_b) is then calculated according to following equations:

$$R_b = \frac{u_{ext}}{u_{in}} \quad (3),$$

$$u_{ext} = \sqrt{\frac{\sum_{i=1}^{n} [(h_i - h_{ref})/u_i]^2}{(n-1)\sum_{i=1}^{n} u^{-2}(h_i)}} \quad (4),$$

$$u_{in} = \sqrt[2]{(n-1)\sum_{i=1}^{n} u^{-2}(h_i)} \quad (5),$$

Method S3 Selection of the most appropriate estimation method for the mean DNA copy number concentration obtained in the inter-laboratory study.

For the mean DNA copy number concentration with each material, the measurement uncertainties and degrees of freedom were reported from five dPCR instruments from three laboratories. Based on the CCQM guidelines (CCQM, 2013), several criteria were considered to facilitate the selection of the most appropriate method for estimation of the mean DNA copy number concentrations and the corresponding measurement uncertainties. The main two criteria were: (i) presence of extreme results (i.e., outliers); and (ii) consistency of the mean DNA copy number concentration between instruments (i.e., absence of over-dispersion), with corresponding measurement uncertainties taken into account (CCQM, 2013). For each HCMV test material, no outliers were determined using Grubbs tests (Table S14), and therefore robust estimators were not necessarily needed. Additionally, for

datasets of fewer than seven values, robust estimators are not recommended (CCQM, 2013). With each material, preliminary graphical inspection, chi-square tests (Table S15) and calculated Birge ratios (WVM units, 2.38; gDNA units, 2.30) suggested minor over-dispersion (i.e., inconsistency) of the results. This finding indicated that the measurement uncertainties with the corresponding mean DNA copy number concentrations did not fully account for the observed variability between the dPCR instruments. Based on these two criteria, the most suitable estimators would be the unweighed Arithmetic mean, the weighed Mandel-Paule estimator, and the weighed Vangel-Ruhkin estimator. Considering the moderate variability between the reported relative expanded measurement uncertainties (WVM units, 8%-28%; gDNA units, 6%-18%) and the limited degrees of freedom for the reported results (k=2.11 or k=2.2), the weighed Vangel-Ruhkin estimator was selected as the estimation method of choice.

Table S1 Data for the Biomark 37K array from Laboratory 1

Material	Experiment number[a]	Unit	Gravimetric dilution	Number of positive chambers	Number of chambers analysed	Mean partition occupancy	Copy number (cp/µL)
WVM	1	W1	1×	475	770	0.96	3262
WVM	1	W1	1×	515	770	1.11	3758
WVM	1	W2	1×	512	770	1.09	3718
WVM	1	W2	1×	517	770	1.11	3785
WVM	1	W3	1×	573	770	1.36	4635
WVM	1	W3	1×	551	770	1.26	4275
WVM	2	W1	2×[b]	375	770	0.67	4539
WVM	2	W1	2×[b]	333	770	0.57	3852
WVM	2	W2	2×[b]	336	770	0.57	3899
WVM	2	W2	2×[b]	361	770	0.63	4303
WVM	2	W3	1.97×[b]	410	770	0.76	5115
WVM	2	W3	1.97×[b]	350	770	0.61	4078
gDNA	1	G1	1×	175	770	0.26	877
gDNA	1	G1	1×	191	770	0.29	969
gDNA	1	G2	1×	233	770	0.36	1225
gDNA	1	G2	1×	212	770	0.32	1095
gDNA	1	G3	1×	203	770	0.31	1041
gDNA	1	G3	1×	207	770	0.31	1065
gDNA	2	G1	1×	209	770	0.32	1077
gDNA	2	G1	1×	200	770	0.30	1023
gDNA	2	G2	1×	217	770	0.33	1126
gDNA	2	G2	1×	210	770	0.32	1083
gDNA	2	G3	/[c]	/[c]	/[c]	/[c]	/[c]
gDNA	2	G3	/[c]	/[c]	/[c]	/[c]	/[c]

[a] Only two experiments were performed due to technical problems with experiment 3.

[b] Dilutions were performed due to initial lost of certain aliquot volumes.

[c] Due to an error in preparation of a specific aliquot, these data were not valid.

Table S2 Data for the QX100 from Laboratory 1

Material	Experiment number	Unit	Gravimetric dilution	Number of positive chambers	Number of chambers analysed	Mean partition occupancy	Copy number (cp/µL)
WVM	1	W1	11.00×	1674	16402	0.11	3550
WVM	1	W1	11.00×	1546	15835	0.10	3387
WVM	1	W2	10.88×	1677	14377	0.12	4047
WVM	1	W2	10.88×	1825	15319	0.13	4139
WVM	1	W3	9.94×	1680	13642	0.13	3919
WVM	1	W3	9.94×	1766	13843	0.14	4070
WVM	2	W1	9.77×	1782	15985	0.12	3463
WVM	2	W1	9.77×	1735	15380	0.12	3507
WVM	2	W2	10.02×	1611	13803	0.12	3730
WVM	2	W2	10.02×	1637	13963	0.12	3748
WVM	2	W3	9.56×	1998	15410	0.14	3980
WVM	2	W3	9.56×	1754	14153	0.13	3792
WVM	3	W1	10.07×	1694	15403	0.12	3519
WVM	3	W1	10.07×	1629	15173	0.11	3431
WVM	3	W2	11.08×	1583	14859	0.11	3743
WVM	3	W2	11.08×	1572	14498	0.11	3814
WVM	3	W3	10.54×	1926	15895	0.13	4081
WVM	3	W3	10.54×	1812	14793	0.13	4129
gDNA	1	G1	7.80×	650	15029	0.04	1035
gDNA	1	G1	7.80×	552	14317	0.04	920
gDNA	1	G2	8.40×	417	14333	0.03	743
gDNA	1	G2	8.40×	354	13749	0.03	657
gDNA	1	G3	9.80×	444	13872	0.03	956
gDNA	1	G3	9.80×	483	14115	0.03	1023
gDNA	2	G1	9.77×	464	13529	0.03	1023
gDNA	2	G1	9.77×	438	15027	0.03	867
gDNA	2	G2	9.77×	496	14524	0.03	1018
gDNA	2	G2	9.77×	527	14929	0.04	1053
gDNA	2	G3	9.92×	451	13521	0.03	1009
gDNA	2	G3	9.92×	557	16773	0.03	1005
gDNA	3	G1	10.02×	467	14811	0.03	963
gDNA	3	G1	10.02×	393	13523	0.03	886
gDNA	3	G2	10.83×	385	14308	0.03	886
gDNA	3	G2	10.83×	455	14299	0.03	1050
gDNA	3	G3	10.28×	473	15346	0.03	966
gDNA	3	G3	10.28×	476	15862	0.03	940

Table S3 Data for the Biomark 37K array from Laboratory 2

Material	Experiment number	Unit	Gravimetric dilution	Number of positive chambers	Number of chambers analysed	Mean partition occupancy	Copy number (cp/µL)
WVM	1	W4	1×	433	770	0.83	2810
WVM	1	W4	1×	425	770	0.80	2730
WVM	1	W5	1×	392	770	0.71	2419
WVM	1	W5	1×	396	770	0.72	2455
WVM	1	W6	1×	224	770	0.34	1169
WVM	1	W6	1×	310	770	0.52	1752
WVM	2	W4	1×	449	770	0.87	2975
WVM	2	W4	1×	486	770	1.00	3392
WVM	2	W5	1×	392	770	0.71	2419
WVM	2	W5	1×	253	770	0.40	1355
WVM	2	W6	1×	338	770	0.58	1965
WVM	2	W6	1×	348	770	0.60	2045
WVM	3	W4	1×	485	770	0.99	3380
WVM	3	W4	1×	460	770	0.91	3094
WVM	3	W5	1×	497	770	1.04	3526
WVM	3	W5	1×	492	770	1.02	3464
WVM	3	W6	1×	411	770	0.76	2595
WVM	3	W6	1×	380	770	0.68	2313
gDNA	1	G4	1×	151	770	0.22	742
gDNA	1	G4	1×	155	770	0.22	764
gDNA	1	G5	1×	151	770	0.22	742
gDNA	1	G5	1×	168	770	0.25	837
gDNA	1	G6	1×	231	770	0.36	1213
gDNA	1	G6	1×	217	770	0.33	1126
gDNA	2	G4	1×	208	770	0.31	1071
gDNA	2	G4	1×	201	770	0.30	1029
gDNA	2	G5	1×	205	770	0.31	1053
gDNA	2	G5	1×	185	770	0.27	934
gDNA	2	G6	1×	191	770	0.29	969
gDNA	2	G6	1×	127	770	0.18	613
gDNA	3	G4	1×	199	770	0.30	1017
gDNA	3	G4	1×	200	770	0.30	1023
gDNA	3	G5	1×	213	770	0.32	1101
gDNA	3	G5	1×	182	770	0.27	917
gDNA	3	G6	1×	200	770	0.30	1023
gDNA	3	G6	1×	194	770	0.29	987

Table S4 Data for the QX100 from Laboratory 2. Measurements in bold were excluded from further analysis due to increased droplet fluorescence

Material	Experiment number	Unit	Gravimetric dilution	Number of positive chambers	Number of chambers analysed	Mean partition occupancy	Copy number (cp/µL)
WVM	1	W4	10.19×	1655	17462	0.10	3042
**WVM**	**1**	**W4**	**10.19×**	**1620**	**12693**	**0.14**	**4171**
WVM	1	W5	10.50×	1231	15788	0.08	2555
WVM	1	W5	10.50×	1093	15103	0.08	2364
WVM	1	W6	10.41×	1183	15947	0.08	2405
WVM	1	W6	10.41×	1083	14517	0.08	2419
WVM	2	W4	10.08×	1683	15881	0.11	3384
WVM	2	W4	10.08×	1665	15556	0.11	3420
WVM	2	W5	10.56×	1350	15518	0.09	2881
**WVM**	**2**	**W5**	**10.56×**	**1253**	**11580**	**0.11**	**3625**
WVM	2	W6	10.61×	1217	13526	0.09	2998
WVM	2	W6	10.61×	1162	14504	0.08	2655
WVM	3	W4	12.12×	1323	14699	0.09	3427
WVM	3	W4	12.12×	1394	15162	0.10	3504
WVM	3	W5	10.61×	1138	15355	0.08	2449
WVM	3	W5	10.61×	1082	14802	0.08	2414
WVM	3	W6	10.35×	1345	14312	0.10	3062
WVM	3	W6	10.35×	1336	14947	0.09	2905
gDNA	1	G4	10.08×	529	16269	0.03	999
**gDNA**	**1**	**G4**	**10.08×**	**486**	**13122**	**0.04**	**1140**
gDNA	1	G5	9.61×	602	16308	0.04	1084
**gDNA**	**1**	**G5**	**9.61×**	**516**	**13049**	**0.04**	**1162**
gDNA	1	G6	10.45×	589	16086	0.04	1169
gDNA	1	G6	10.45×	502	15788	0.03	1012
gDNA	2	G4	10.21×	470	12720	0.04	1152
gDNA	2	G4	10.21×	553	14948	0.04	1153
**gDNA**	**2**	**G5**	**10.64×**	**480**	**11941**	**0.04**	**1308**
gDNA	2	G5	10.64×	591	16251	0.04	1181
gDNA	2	G6	10.42×	566	15695	0.04	1147
gDNA	2	G6	10.42×	495	15690	0.03	1001
gDNA	3	G4	10.74×	588	16411	0.04	1175
gDNA	3	G4	10.74×	569	15948	0.04	1170
gDNA	3	G5	10.07×	561	15480	0.04	1114
gDNA	3	G5	10.07×	566	15325	0.04	1136
gDNA	3	G6	9.86×	620	16185	0.04	1154
gDNA	3	G6	9.86×	583	16469	0.04	1065

Table S5 Data for the Biomark 37K array from Laboratory 3

Material	Experiment number	Unit	Gravimetric dilution	Number of positive chambers	Number of chambers analysed	Mean partition occupancy	Copy number (cp/µL)
WVM	1	W7	1×	540	770	1.21	4090
WVM	1	W7	1×	440	770	0.85	2868
WVM	1	W8	1×	485	770	0.99	3364
WVM	1	W8	1×	486	770	1.00	3376
WVM	1	W9	1×	483	770	0.99	3340
WVM	1	W9	1×	447	770	0.87	2940
WVM	2	W7	1×	503	770	1.06	3585
WVM	2	W7	1×	480	770	0.98	3305
WVM	2	W8	1×	422	770	0.79	2688
WVM	2	W8	1×	438	770	0.84	2847
WVM	2	W9	1×	450	770	0.88	2972
WVM	2	W9	1×	447	770	0.87	2940
WVM	3	W7	1×	508	770	1.08	3649
WVM	3	W7	1×	512	770	1.09	3701
WVM	3	W8	1×	464	770	0.92	3123
WVM	3	W8	1×	486	770	1.00	3376
WVM	3	W9	1×	494	770	1.03	3473
WVM	3	W9	1×	490	770	1.01	3424

Table S6 Data for the Quant Studio 3D from Laboratory 4

Material	Experiment number[a]	Unit	Gravimetric dilution	Number of positive chambers	Number of accepted chambers[a]	Total number of accepted chambers	Meean partition occupancy	Copy number (cp/µL)
gDNA	1	G7	2.32×	5371	18353	18565	0.35	991
gDNA	1	G7	2.25×	5424	17809	17988	0.36	1012
gDNA	1	G8	2.28×	5167	18701	18823	0.32	911
gDNA	1	G8	2.24×	5386	18532	18634	0.34	949
gDNA	1	G9	2.24×	4905	18314	18923	0.31	864
gDNA	1	G9	2.25×	5017	18166	18225	0.32	900
gDNA	2	G7	2.27×	5465	18722	18770	0.35	967
gDNA	2	G7	2.26×	5268	18319	18422	0.34	946
gDNA	2	G8	2.29×	4998	17994	18702	0.33	919
gDNA	2	G8	2.24×	5318	17915	17975	0.35	976
gDNA	2	G9	2.25×	5130	17937	17983	0.34	936
gDNA	2	G9	2.27×	5098	18961	19382	0.31	878
gDNA	3	G7	2.27×	5609	18422	18558	0.36	1021
gDNA	3	G7	2.26×	5524	18610	18678	0.35	984
gDNA	3	G8	2.28×	5219	18700	18867	0.33	924
gDNA	3	G8	2.28×	5698	18278	18410	0.37	1051
gDNA	3	G9	2.28×	4873	18914	19579	0.30	838
gDNA	3	G9	2.29×	5190	19105	19229	0.32	896

[a] after application of 'colour by quality' threshold

Table S7 Information on the *UL54* assay

**Sequence accession number**	**Amplicon length (bp)**	**Type of oligonucleotide**	**Sequence (5’->3’)**	**Source**	**Purification type**
AY422361	72	Forward primer	GGCCGTTACTGTCTGCAGGA	Eurofins Scientific	Not provided by manufacturer
		Reverse primer	GGCCTCGTAGTGAAAATTAATGGT	Eurofins Scientific	Not provided by manufacturer
		Probe	FAM-CCGTATTGGTGCGCGATCTGTTCAA-BHQ	Eurofins Scientific	HPLC

Table S8 dMIQE checklist for authors, reviewers and editors

Item	Importance[a]	Checklist
**Experimental design**		
Definition of experimental and control groups	E	Materials and methods
Number within each group	E	Materials and methods
Assay carried out by core laboratory or investigator's laboratory?	D	Materials and methods
Power analysis	D	N/A
**Sample**		
Description	E	Materials and methods
Volume or mass of sample processed	E	Materials and methods
Microdissection or macrodissection	E	N/A
Processing procedure	E	Materials and methods
If frozen, how and how quickly?	E	N/A
If fixed, with what, and how quickly?	E	N/A
Sample storage conditions and duration (especially for formalin-fixed, paraffin-embedded samples)	E	Materials and methods
**Nucleic-acid extraction**		
Quantification instrument/ method	E	Materials and methods
Storage conditions (temperature, concentration, duration, buffer)	E	Materials and methods
DNA or RNA quantification	E	Supplementary Method S1<br>Results and Discussion
Quality/ integrity, instrument/method (e.g., RNA integrity/ R quality index) and trace or 3':5'	E	N/A
Template structural information	E	N/A
Template modification (e.g., digestion, sonication, preamplification)	E	N/A
Template treatment (initial heating or chemical denaturation)	E	N/A
Inhibition dilution or spike	E	Published previously (Pavšič et al., 2016)
DNA contamination assessment of RNA sample	E	N/A
Details of DNase treatment where performed	E	N/A
Manufacturer of reagents used and catalogue number	D	Materials and methods
Storage of nucleic acid (temperature, concentration, duration, buffer)	E	Materials and methods<br>Supplementary Method S1
**dPCR target information**		
Sequence accession number	E	Supplementary Table S7
Amplicon location	D	N/A
Amplicon length	E	Supplementary Table S7

In-silico specificity screen (e.g., BLAST)	E	Published previously (Pavšič et al., 2016)
Pseudogenes, retropseudogenes, or other homologues?	D	N/A
Sequence alignment	D	N/A
Secondary structure analysis of amplicon and GC content	D	N/A
Location of each primer by exon or intron (if applicable)	E	N/A
Where appropriate, which splice variants are targeted?	E	N/A
**dPCR oligonucleotides**		
Primer sequences and/or amplicon context sequence	E	Supplementary Table S7
RTPrimerDB (real-time PCR primer and probe database) identification number	D	N/A
Probe sequences	D	Supplementary Table S7
Location and identity of any modifications	E	N/A
Manufacturer of oligonucleotides	D	Supplementary Table S7
Purification method	D	Supplementary Table S7
**dPCR protocol**		
Complete reaction conditions	E	Materials and methods
Reaction volume and amount of cDNA/DNA	E	Materials and methods
Primer, (probe), Mg^{2+} and dNTP concentrations	E	Materials and methods, some not available due to commercial kit
Polymerase identity and concentration	E	N/A due to commercial kit
Buffer/ kit identity and manufacturer	E	Materials and methods
Exact chemical constitution of the buffer	D	N/A due to commercial kit
Additives (e.g., SYBR Green I, DMSO)	E	N/A due to commercial kit
Plates/tubes catalogue number and manufacturer	D	N/A
Complete thermocycling parameters	E	Materials and methods
Reaction set-up (manual/ robotic)	D	Materials and methods Supplementary material
Gravimetric or volumetric dilutions (manual/ robotic)	D	Gravimetric dilutions (manual)
Total PCR volume prepared	D	Supplementary Table S11
Partition number	E	Supplementary Tables S1, S2, S3, S4, S5, S6, S11
Individual partition volume	E	Supplementary Table S11
Total volume of the partitions measured (effective reaction size)	E	Supplementary Table S11
Partition volume variance/SD	D	Supplementary Table S11
Comprehensive details and appropriate use of	E	Materials and methods

controls		
Manufacturer of dPCR instrument	E	Materials and methods
**dPCR validation**		
Optimisation data for the assay	D	Published previously (Pavšič et al., 2016)
Specificity (e.g., when measuring rare mutations, pathogen sequences)	E	Published previously (Pavšič et al., 2016)
Limit of detection of calibration control	D	N/A
If multiplexing, comparison with singleplex assays	E	N/A
**Data analysis**		
Mean copies per partition (λ or equivalent)	E	Supplementary Tables S1, S2, S3, S4, S5, S6
dPCR analysis programme (source, version)	E	Materials and methods<br>Supplementary Tables S9, S10
Outlier identification and disposition	E	Materials and methods<br>Supplementary Table S12
Results of NTCs	E	Materials and methods
Examples of positive(s) and negative experimental data as supplemental data	E	Supplementary Figure S1, S6, S7
Where appropriate, justification of number and choice of reference genes	E	N/A
Where appropriate, description of normalisation method	E	N/A
Number and concordance of biological replicates	D	N/A
Number and stage (RT or qPCR) of technical replicates	E	Materials and methods
Repeatability (intra-assay variation)	E	Figures 2, 3
Reproducibility (e.g., inter-assay/ user/ laboratory variation)	D	Figures 2, 3, 4
Experimental variance or CI	E	N/A
Statistical methods for analysis	E	Materials and methods
Data submission using real-time PCR data mark-up language	D	N/A

[a] D, desirable information; E, essential information

N/A, not applicable

Table S9 Analysis parameters using the Biomar HD Data Collection Software (Fluidigm) on the Biomark system

Laboratory	Software version	Ct threshold method	Ct threshold	Quality threshold	Cq range	Baseline correction method
1 (NIB)	3.1.4	User (Manual)	0.09	0.35	20-45	Linear
2 (Directorate F)	4.1.2	User (Global)	0.1	0.3	20-45	Linear
3 (LGC)	4.0.1	User (Global)	0.1	0.65	20-40	Linear

Table S10 Analysis parameters using the QuantaSoft analysis software (Bio-Rad) on the QX100 system

Laboratory	Software version	Threshold method	Threshold value
1 (NIB)	1.3.2.0	Manual	4000 (experiments 1, 3), 7500 (experiment 2)
2 (Directorate F)	1.6	Manual	3000

Table S11 Technical information for the three platforms used for both of the materials and by all four of the laboratories

**Information**	**QX100**	**Biomark (37 K array)**	**QuantStudio 3D**
Theoretical number of partitions	20,000	770	20,000
Number of partitions analysed	Lab 1: 14,770 (±870)<br>Lab 2: 15,140 (± 1430)	770	18,318 (±400)
Effective reaction size (µL)	Lab 1: 12.3 (±0.7)<br>Lab 2: 12. 6 (±1.2)	0.647	14.8 (±0.32)
Total reaction volume (µL)	20	4	15
DNA volume per total reaction volume (µL)	8	2.8	6.75
Partition volume (nL)	0.834	0.847	0.809: updated volume (initial volume of chip was 0.865)

Table S12 Grubbs test for outliers within a single instrument. The Grubbs test was performed in R studio using the 'grubbs.test' function and taking into account all of the measured DNA copy number concentrations within a single dPCR instrument. If the measured DNA copy number concentration was determined to be an outlier ($p < 0.05$), the remaining measurements were reanalysed in another round of Grubbs test. The outliers that were defined are shown in bold

Material	Laboratory	Platform	Candidate for outlier	Outlier (p-value)
WVM	1	Biomark	5155	0.15
WVM	1	QX100	3387	1.0
WVM	2	Biomark	1169	0.36
WVM	2	QX100	3503	0.92
WVM	3	Biomark	4090	0.14
gDNA	1	Biomark	876	0.14
**gDNA**	**1**	**QX100**	**657**	**0.02**
**gDNA**	**1**	**QX100**	**743**	**0.02**
gDNA	1	QX100	867	0.52
gDNA	2	Biomark	613	0.18
gDNA	2	QX100	999	0.49
gDNA	4	QuantStudio 3D	1051	0.43

Table S13 DNA copy number concentrations and coefficient of variability for the experiments on the different instruments. In each experiment, three units were measured in duplicate. P-value denotes statistical significance of differences between experiments

HCMV test material	Lab.	Platform	Experiment 1		Experiment 2		Experiment 3		ANOVA
			Mean DNA copy number (cp/µL)	CV (%)	Mean DNA copy number (cp/µL)	CV (%)	Mean DNA copy number (cp/µL)	CV (%)	p-value
WVM	1	Biomark	3906	12	4298	11	/	/	0.19
WVM	1	QX100	3852	8	3703	5	3786	8	0.64
WVM	2	Biomark	2223	29	2358	31	3062	16	0.08
WVM	2	QX100	2557	11	3068	11	2960	16	0.11
WVM	3	Biomark	3330	13	3056	11	3458	6	0.14
gDNA	1	Biomark	1045	11	1077	4	/	/	0.67
gDNA	1	QX100	983	6	996	7	948	6	0.42
gDNA	2	Biomark	904	23	945	18	1011	6	0.52
gDNA	2	QX100	1065	7	1127	6	1136	4	0.36
gDNA	4	QuantStudio 3D	938	6	937	4	952	8	0.89

Table S14 Grubbs test for outliers among the different dPCR instruments. The Grubbs test was performed in R studio using the 'grubbs.test' function and taking into account mean DNA copy number concentrations from the five dPCR instruments. The criterion for an outlier was $p < 0.05$

Material	Candidate for outlier	p-value
WVM	4101	0.50
gDNA	1114	0.27

Table S15 Chi-squared test for mutual consistency between the instruments. The Chi-squared test was performed in Excel 2007 using the equations from the CCQM report (CCQM, 2013). The reported mean DNA copy number concentrations are mutually consistent if the calculated chi-squared value is lower than the chi-squared value for the 95% confidence interval

Material	Calculated chi-squared	95% confidence interval for chi-squared
WVM	22.7	9.49
gDNA	21.2	9.49

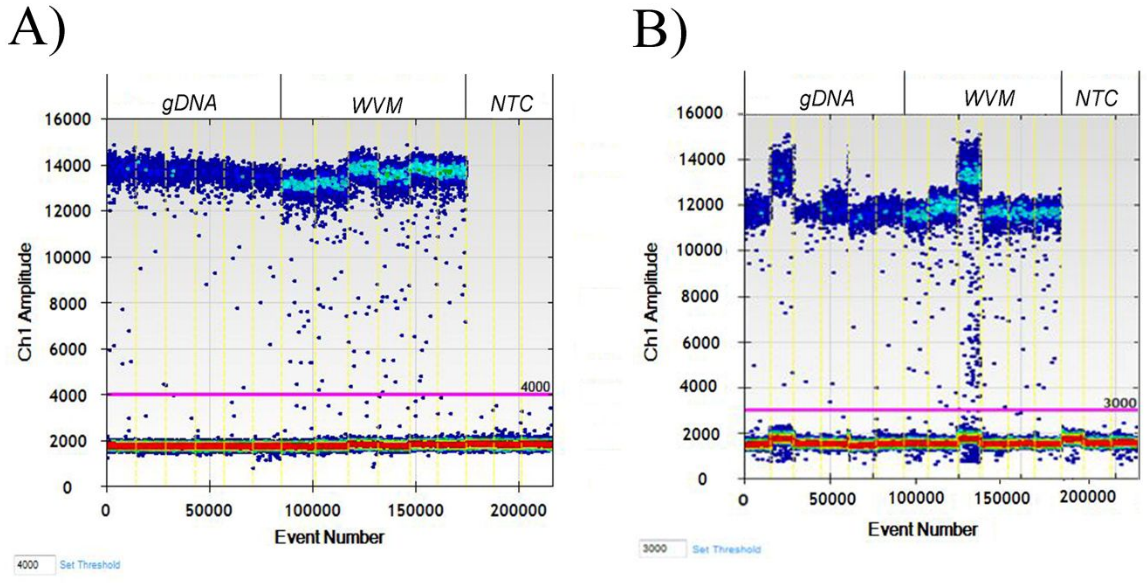

Fig. S1 Examples of positive and negative reactions on the QX100 system over two laboratories. Positive and negative reactions from a single experiment from Laboratory 1 **(A)** and Laboratory 2 **(B)** are shown. In Laboratoy 2 (B), second column of gDNA and third column of WVM are the exaples of increased droplet fluorescence, which were consequently excluded from further analyses

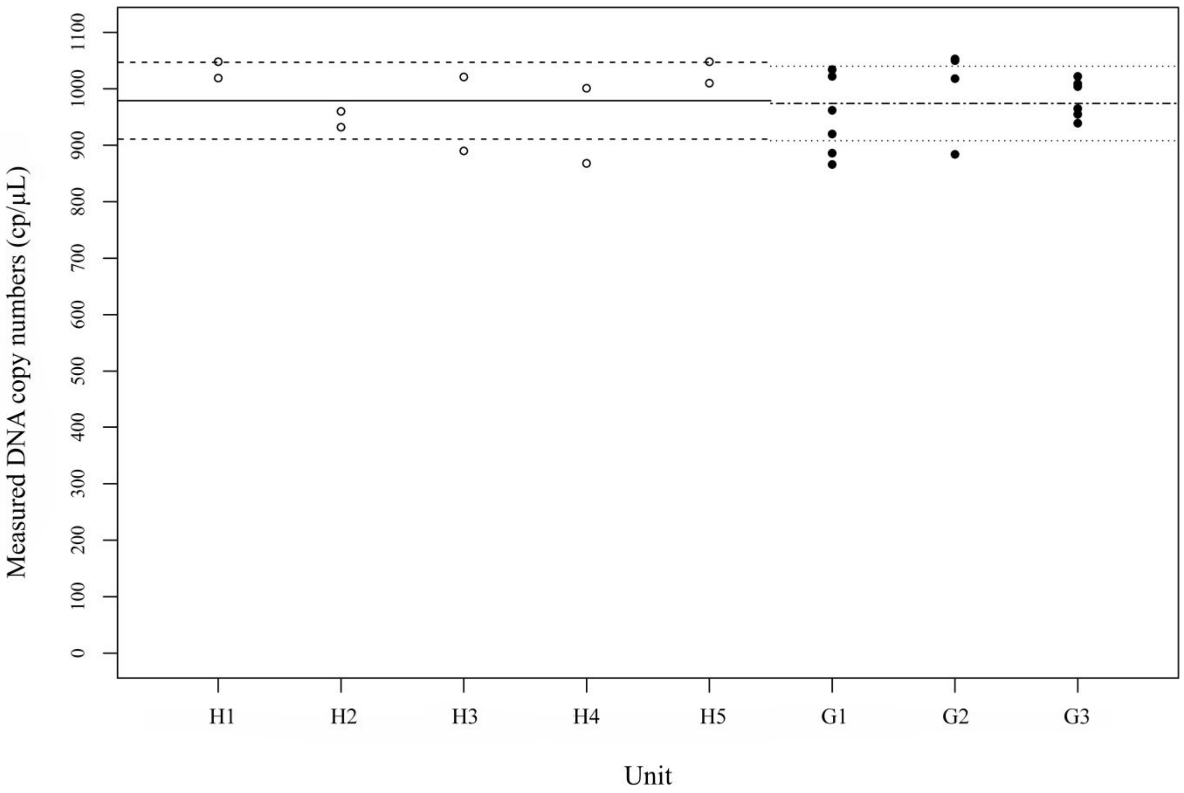

Fig. S2 Homogeneity and stability of the gDNA material (i.e., in the gDNA units). Homogeneity of the gDNA test sample batch was assessed by analysis of five units of gDNA (i.e., gDNA units H1-H5) in duplicate on the QX100 system in Laboratory 1. The stability was tested by analysing three gDNA units previously stored for four months at -20 °C (i.e., gDNA units G1-G3). Each of these three gDNA units was tested in duplicate over three consecutive experiments as part of the inter-laboratory study in Laboratory 1. Open circles represent the measured concentrations of the gDNA units (H1-H5) during the homogeneity study, and the filled circles depict the measured values of the three gDNA units (G1-G3) as a part of the inter-laboratory assessment. Each H unit was measured in duplicate (n=2), whereas each G unit was measured in duplicate over three experiments (n=6). The mean DNA concentration of the H units is depicted as the full line, and the two dashed lines show the expanded measurement uncertainty (95% confidence interval). The mean DNA concentration of the G1-G3 units is depicted as a dashed and dotted line, with the dotted lines showing the measurement uncertainty (95% confidence interval). For the G units, the outliers determined by the Grubbs tests were omitted here

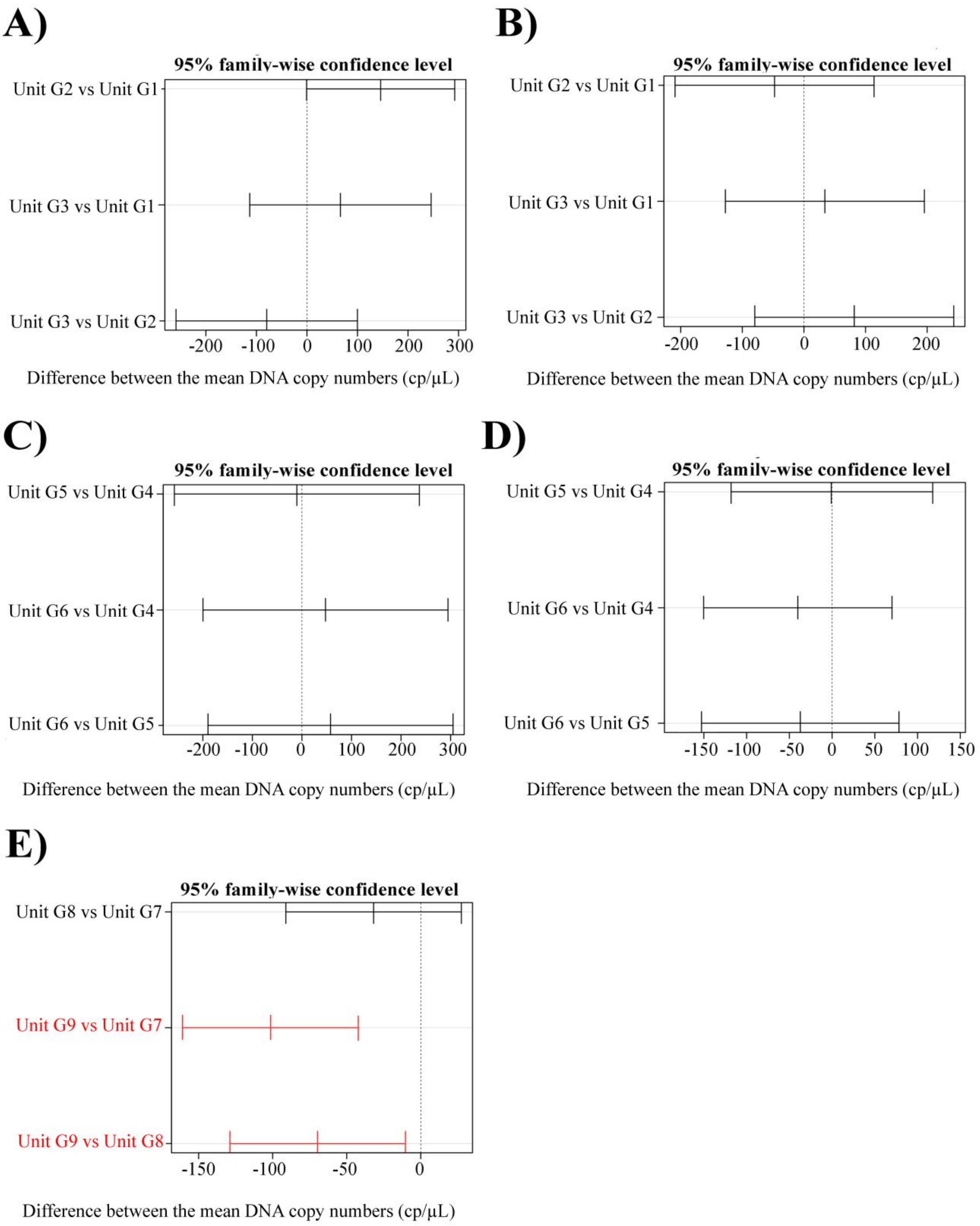

Fig. S3 Tukey tests for statistically significant differences between the gDNA units (e.g., Figure 1B: Laboratory 1, units G1-G3; Laboratory 2, units G4-G7; Laboratory 4, units G7-G9) in terms of the mean DNA copy number concentrations. For Laboratory 1, the Biomark 37K array **(A)** and the QX100 system **(B)** are shown. For Laboratory 2, the Biomark 37K array **(C)** and the QX100 **(D)** are shown. For Laboratory 4, the QuantStudio 3D is shown **(E)**. gDNA unit pairs in red indicate statistically significant differences

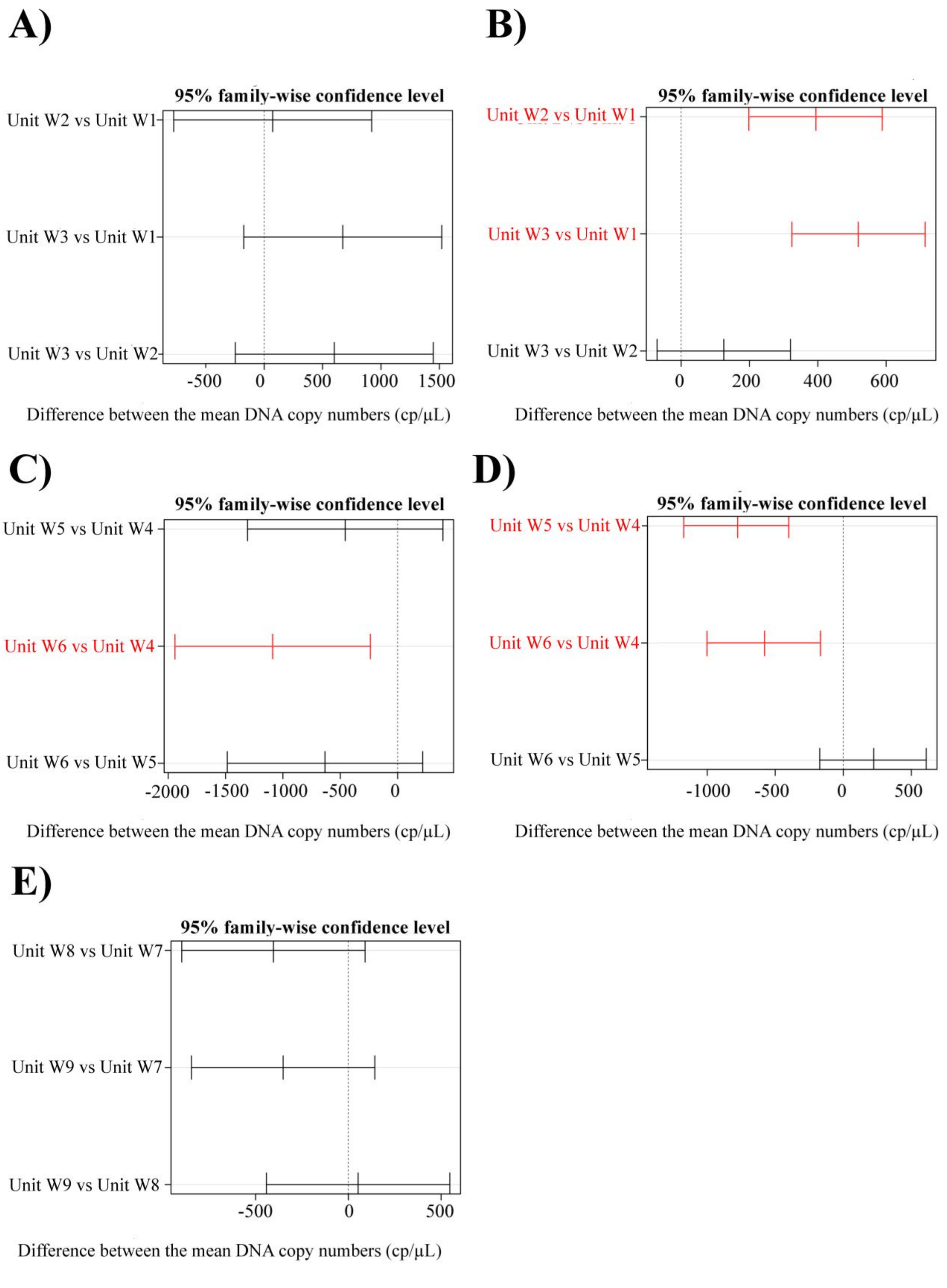

Fig. S4 Tukey tests for statistically significant differences between the WVM units (e.g., Figure 1A: Laboratory 1, units W1-W3; Laboratory 2, units W4-W7; Laboratory 3, units W7-W9) in terms of the mean DNA copy number concentration. For Laboratory 1, the Biomark 37K array **(A)** and the QX100 system **(B)** are shown. For Laboratory 2, the Biomark 37K array **(C)** and the QX100 **(D)** are shown. For Laboratory 3, the Biomark system **(E)** is shown. WVM unit pairs in red indicate statistically significant differences

A)

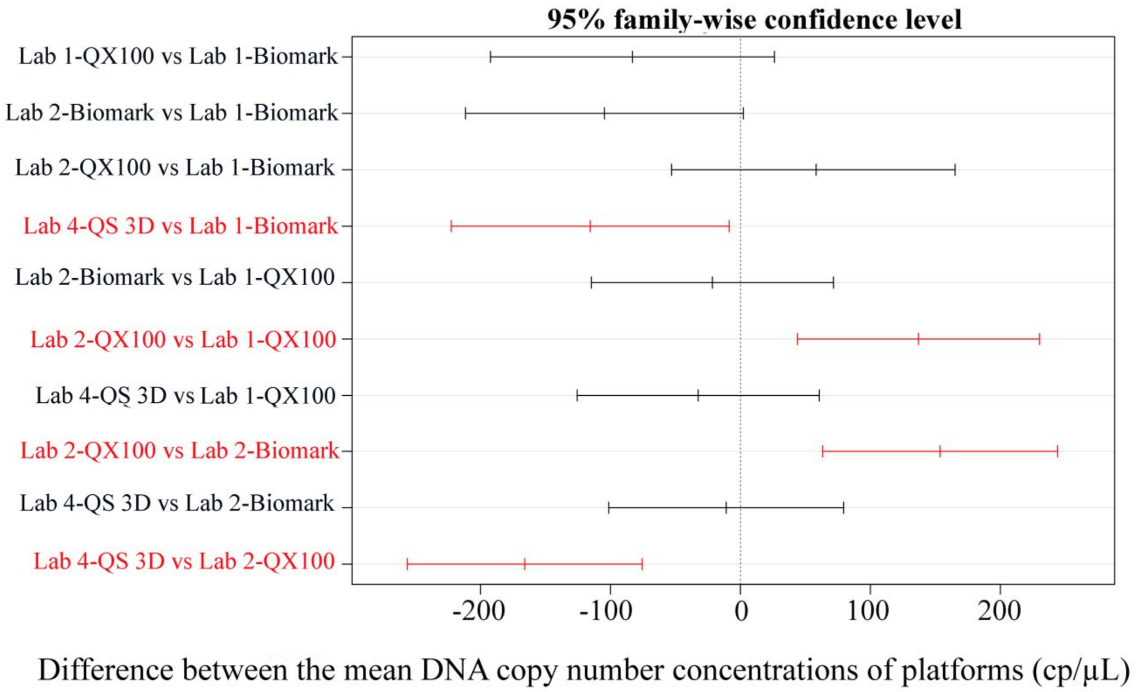

B)

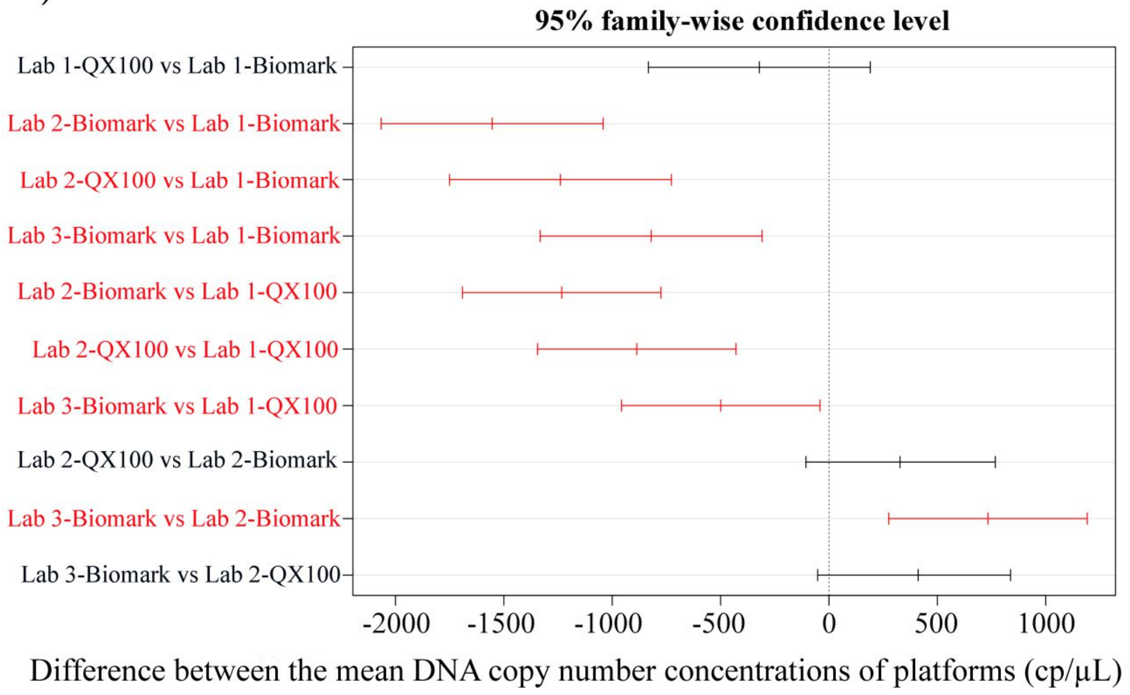

Fig. S5 Tukey tests for statistically significant differences between the mean DNA copy number concentrations obtained from the different instruments. The statistically significant differences obtained with the gDNA units **(A)** and WVM units **(B)** are shown. Intrument pairs in red indicate statistically significant differences in the mean DNA copy numbers observed between the particular instruments

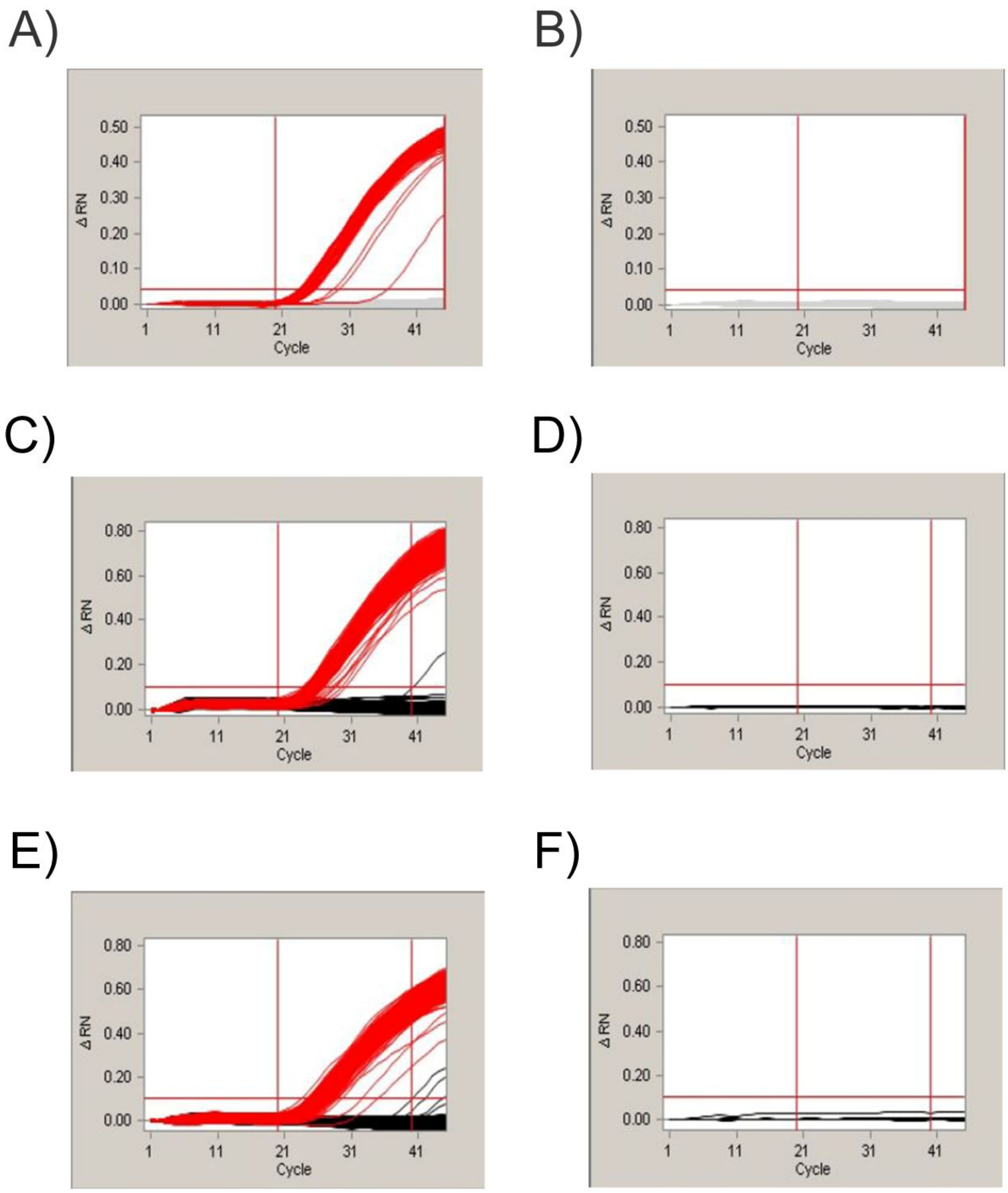

Fig. S6 Examples of positive and negative reactions performed on the Biomark 37K array over three laboratories. **(A)** Positive reaction in Laboratory 1. **(B)** Negative reaction in Laboratory 1. **(C)** Positive reaction in Laboratory 2. **(D)** Negative reaction in Laboratory 2. **(E)** Positive reaction in Laboratory 3. **(F)** Negative reaction in Laboratory 3.

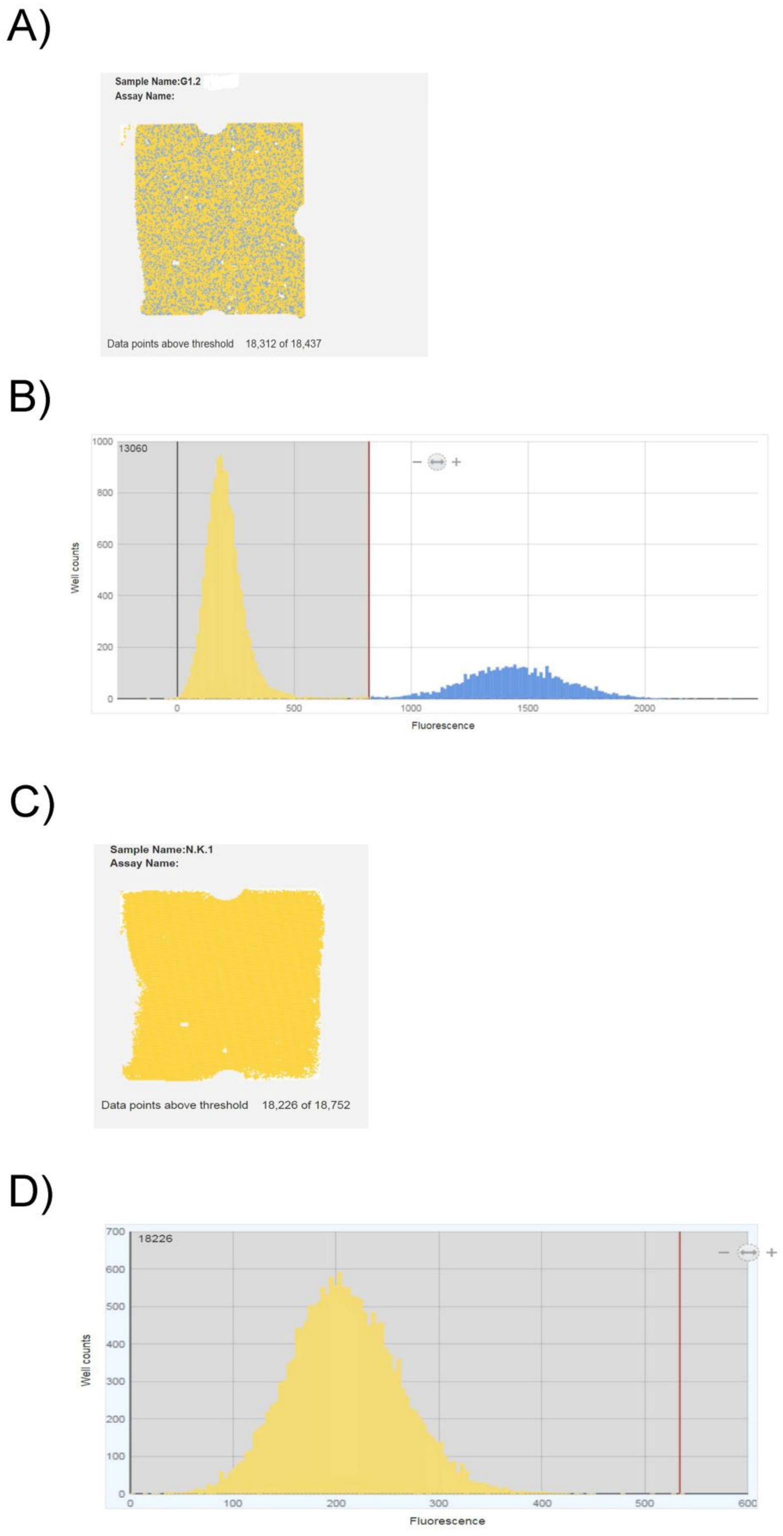

Fig. S7 Examples of positive and negative reactions on the QuantStudio 3D system in Laboratory 4. **(A)** Local distribution of accepted droplets from a positive sample tested on a single achip. **(B)** Fluorescence intensitites of every accepted droplet, from (A). **(C)** Local distribution of accepted droplets from a negative control. **(D)** Fluorescence intensities of every accepted droplet, from (C)

References

CCQM. (2013). CCQM Guidance note: Estimation of a concensus KCRV and associated degrees of equivalence. Retrieved from http://www.bipm.org/cc/CCQM/Allowed/19/CCQM13-22_Consensus_KCRV_v10.pdf
